# A Prominent Pro‐Inflammatory Phenotype Is Observed in Replication and Stress‐Induced Senescent Mast Cells

**DOI:** 10.1111/acel.70186

**Published:** 2025-08-28

**Authors:** A. Ibarra‐Sánchez, I. Madera‐Salcedo, D. Esparza‐Reyes, P. Mendoza‐Montiel, J. Padilla, C. González‐Espinosa

**Affiliations:** ^1^ Pharmacobiology Department Center for Research and Advanced Studies (Cinvestav) of the National Polytechnic Institute (IPN) Mexico City Mexico; ^2^ Center for Research in Aging, Cinvestav Mexico City Mexico; ^3^ Immunology and Rheumatology Department National Institute of Medical Sciences and Nutrition Salvador Zubirán Mexico City Mexico; ^4^ Microbiology and Parasitology Department, Faculty of Medicine National Autonomous University of Mexico Mexico City Mexico

**Keywords:** inflammaging, innate immunity, mast cells, senescence

## Abstract

Mast cells (MC) are long‐lived important immune effectors that control inflammation, allergies, and innate immunity reactions, but the expression of specific markers in replicative and stress‐induced senescence in this cell type, together with its relevance in vivo, has not been described. Here, bone marrow‐derived MCs (BMMC) were generated from young C57BL6/J mice and kept in culture for a long time or treated with the well‐known stressor bacterial lipopolysaccharide (LPS) to promote replicative and stress‐induced senescence, respectively. Changes in size, granularity, and expression of p16^INK4A^ and p21^CIP1/WAF1^, together with cell cycle arrest and senescence‐associated‐β‐Galactosidase (SA‐β‐Gal) activity, were observed after 12 weeks in culture, with minimal changes in cell viability but important modifications in cell metabolism. Senescence‐associated secretory phenotype (SASP) included IL‐23, IL‐6, and VEGF, among other cytokines and chemokines. Maximal FcεRI and TLR4‐dependent cell activation was diminished by replicative senescence in BMMC. Stress‐induced senescence produced cell cycle arrest, increased β‐Gal expression, and transient high cytokine expression. Utilizing aged MC‐deficient (*c‐Kit* ^*Wsh/Wsh*^) and *c‐Kit* ^*Wsh/Wsh*^ mice reconstituted with MC, the exacerbated cytokine production observed in senescent cells was confirmed in the rapid, canonical MC‐dependent response to acute intraperitoneal LPS administration. Finally, high basal cytokine production was detected in MC purified from chronically LPS‐treated animals. Our data show that (1) senescence markers appear in replication and stress‐induced senescence of MCs; (2) basal and activated effector functions of MC are altered by senescence; and (3) aging is associated with increased MC‐dependent inflammatory responses. Our results show that senescence importantly affects MC function, which could contribute to inflammaging.

## Graphical abstract

Senescent mast cells express canonical senescence markers and develop a pro‐inflammatory phenotype characterized by SASP, increased MAPK and NFκB activation, and altered FcεRI and TLR4‐induced responses. Senescence causes increased mast cell‐dependent inflammaging and innate immunity responses in vivo.

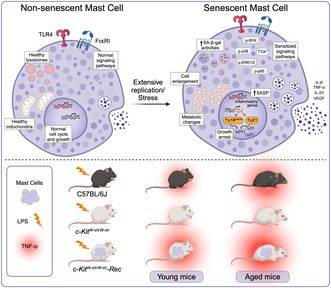

## 1 Introduction

Aging is a process accompanied by a decline in physiological functions due to changes in main regulatory systems in the organism (Campisi 2013; Guo et al. 2022). Increased levels of pro‐inflammatory cytokines due to alterations in immune cell activity are considered the trigger for an age‐related condition known as inflammaging. Immune cell senescence has been proposed as one of the factors that contribute to inflammaging in mice and humans (Z. Liu et al. 2023). The senescent state can occur due to extensive replication or stressful agents (Herranz and Gil 2018) and is characterized by cell‐cycle arrest accompanied by the production of selected pro‐inflammatory mediators that constitute the senescence‐associated secretory phenotype (SASP). At the cellular level, molecular markers of cell senescence are well defined (reviewed in (Hernandez‐Segura et al. 2018)). Those include changes in cell size, expression of the cell cycle regulators p16^INK4A^ (p16) and p21^CIP1/WAF1^ (p21), production of senescence‐associated β‐galactosidase (SA‐βGal), and acquisition of SASP.

Mast cells (MC) constitute an essential member of innate immunity cell types, being not only initiators of acute allergic reactions but also central regulators of tissue inflammation and protective innate immune responses (Malaviya et al. 1996; Echtenacher et al. 1996). Distinct studies have identified this cell lineage as a long‐lived, highly adaptable one, with slow turnover and unique characteristics and functions among the innate immune cells (Chia et al. 2023). Due to their anatomical localization at the frontier between body tissues and the exterior environment, and near to the nerve terminals, one of their main functions seems to be associated with sampling the extracellular milieu to differentially secrete regulatory and proinflammatory mediators able to modulate local and systemic immune, vascular, and neural responses (Frossi et al. 2017). Although distinct studies have reported changes in MC numbers and functions associated with aging (Chatterjee and Gashev 2012; Kundu et al. 2020), the expression of senescence markers in this cell population and its consequences in inflammatory reactions in vivo are not fully described. Due to a lack of information, MC are normally excluded from main reviews on the subject of immune cell senescence despite their relevance in the regulation of numerous pathophysiological processes.

The objective of this work was three‐fold: (1) to characterize the expression of markers of replicative‐ and stress‐induced senescence in a widely used model of MC (bone marrow‐derived mast cells, BMMC); (2) to analyze the consequences of senescence in the capacity of BMMC to respond to FcεRI and TLR4 receptor triggering; and (3) to evaluate the effects of MC senescence in inflammatory reactions in vivo.

## 2 Materials and Methods

### 2.1 Animals

C57BL/6J mice (stock number 000664) and *c‐Kit* ^*Wsh/Wsh*^ (stock number 005051) purchased from The Jackson Laboratory (Bar Harbor, ME) were maintained in controlled animal facilities located in the Center for Research in Aging and the North Campus of the Center for Research and Advanced Studies (Cinvestav). Mice were kept with *ad libitum* access to food and water, with 12–12 h of light. Animals were used following the Mexican official norm for the use of laboratory animals (NOM‐062‐ZOO‐1999). All animal procedures were approved by our Institutional Committee for the Care and Use of Laboratory Animals (CICUAL, protocols No. 384‐24 and 378‐24).

### 2.2 Reagents

Antibodies: anti‐p21^Waf1/Cip1^ (sc‐6246) (Wang et al. 2024), anti‐p16^INK4A^ (sc‐1661) (Dominguez‐Bautista et al. 2021), anti‐p‐p38 (sc‐17852‐R), anti‐β actin (sc‐81178) and anti‐α tubulin (sc‐5286) were from Santa Cruz Biotechnology (Dallas, Texas USA). APC conjugated anti‐mouse FcεRI (17‐5898‐82) antibody was from Thermo Fisher Scientific (Waltham, Massachusetts, USA). PE‐conjugated anti‐mouse TLR4 (145404) and TruStain FcX Plus anti‐mouse CD16/CD32 were from Biolegend (San Diego, CA, USA). Anti‐pERK1/2 (9101), anti‐p NFκB p65 (3033) and anti‐p Src Tyr416 (2101) were from Cell Signaling Technology (Danvers, Massachusetts, USA). HRP‐conjugated goat anti‐rabbit IgG (111‐035‐003) and goat anti‐mouse IgG (115‐035‐003) were from Jackson ImmunoResearch (West Grove, PA). Ghost Dye Red 780 (13‐0865) from Tonbo Cytek Biosciences (San Diego, CA, USA), CellTrace Violet (C34571) from Invitrogen, Thermo Fisher Scientific (Waltham, Massachusetts, USA). Pyronin Y (P9172) and Hoechst 33342 (14533) from Sigma Aldrich (Saint Louis, MO, USA). Bacterial lipopolysaccharide (LPS) from *E. coli* serotype 026:B6 (L8274) was from Sigma Aldrich (Saint Louis, MO, USA). DAPI (4′6′‐diamidino‐2‐phenylindole) was from Thermo Scientific (D1306). ELISA kits were from Peprotech (TNF Cat. No. 900‐K54, IL‐6 900‐K50, VEGF 900‐K99, CCL‐2900‐K126).

### 2.3 Generation of BMMC and IgE Sensitization

Bone marrow from tibias of C57BL/6J (WT) mice was extracted and cultured in RPMI 1640 medium (Sigma‐Aldrich, St. Louis, MO) supplemented with 10% FBS, 100 μg/mL streptomycin, from Invitrogen (Carlsbad, CA). Recombinant mouse IL‐3 (20 ng/mL final concentration, PeproTech, Rocky Hill, NJ) was added to the culture media. BMMC were differentiated by changing the media every 5 to 6 days, collecting non‐adherent cells, and resuspending them in fresh culture medium at a final concentration of one million cells per mL as described (Meurer et al. 2016) for 5 to 6 weeks. This cell preparation was considered mature since it has been largely demonstrated that it shows morphological and functional characteristics of mucosal‐type mast cells, such as electrodense granules and histamine content, accompanied by robust responses in degranulation, migration, and cytokine expression after FcεRI receptor triggering (Meurer et al. 2016; Yu et al. 2018; Chiu and Burrall 1990). Unless it is mentioned, all experiments were performed with cells that were sensitized with 100 ng/mL of anti‐DNP monomeric IgE (clone SPE7) from Sigma‐Aldrich for 18 h at 37°C since this treatment promotes granule formation and maturation of BMMC (Madera‐Salcedo et al. 2013). After sensitization, cells were washed and resuspended (depending on the experiment) in fresh culture medium or Tyrode's BSA buffer (1 mM MgCl_2_, 1.8 mM CaCl_2_, 135 mM NaCl, 5 mM KCl, 5.6 mM glucose, 0.5 g/L BSA and 20 mM HEPES [pH 7.4]) at 37°C. For experiments shown in Figure 5, where cells were chronically exposed to LPS, cells were sensitized before LPS addition, and IgE was not removed during the experiment.

### 2.4 Cell Size and Cell Viability Analysis

Size and granularity, together with the viability of BMMC, were analyzed by flow cytometry. Briefly, one million BMMC were washed with PBS 1X and stained with Ghost Dye Red 780 in PBS for 10 min at 4°C. Then, cells were washed with PBS containing 2% FBS (FACS Buffer) and blocked with 1% anti‐mouse CD16/CD32 in FACS Buffer for 10 min at RT. Subsequently, surface staining with APC‐anti‐FcεRI (1:1000) antibody was performed for 30 min al 4°C. Data were collected using the NovoCyte Quanteon flow cytometer (Agilent) and analyzed with the FlowJo software (v10.8). The gating strategy used is described in Figure S1.

### 2.5 Cell Cycle Analysis

One million BMMC were washed with PBS 1X at RT and stained with Ghost Dye Red 780 for 10 min at 4°C. Then, cells were washed with FACS Buffer (RT) and incubated for 10 min at RT with 1% of TruStain FcX Plus to block Fcγ receptors. Cells were then stained with APC anti‐FcεRI (1:1000) antibody for 30 min at RT and protected from the light. Cells were washed with FACS buffer at RT and resuspended in pre‐warmed (37°C) culture medium at a concentration of 1 × 10^6^ cells/mL. Hoechst dye was added (final concentration 10 μg/mL), mixed, and incubated at 37°C for 45 min in the dark. Then, Pyronin Y was added directly to cells (final concentration 3 μg/mL), mixed, and incubated at 37°C for another 15 min in the dark. Samples were transferred onto ice protected from the light for 15 min. Five hundred cells were acquired in a flow cytometer, NovoCyte Quanteon (Agilent), and analyzed with FlowJo software (v10.8). Granulocytes were gated based on SSC‐A versus FSC‐A, and singlets were selected from the FSC‐A versus FSC‐H and SSC‐A versus SSC‐H dot plots. Dead cells were excluded with Ghost Dye Red780 and MCs were gated on FcεRI^+^ cells (Figure S1).

### 2.6 Proliferation Assay

BMMCs were labeled with CellTrace Violet (CTV) cell proliferation dye as per manufacturer's instructions. Briefly, three million BMMCs were resuspended in 1 mL of warmed PBS and stained with 1 μM CTV. The cells were incubated at 37°C in a darkened water bath for 7 min. Afterward, the dye was quenched by adding 5× volume of medium containing 2% FBS, followed by 5 min of incubation. The cells were pelleted at 300× g for 5 min, resuspended in medium with 2% FBS, and pelleted again. The cells were resuspended in RPMI 1640 supplemented, as previously mentioned, and cultured for 8 days. After this period, the cells were washed and stained for analyses by flow cytometry, as previously mentioned (Figure S1). Cell divisions were analyzed with the proliferation platform of FlowJo v10.8 software.

### 2.7 Metabolic Analysis

Cell metabolism was assessed using a Seahorse XF96 Analyzer (Agilent Technologies). First, an XF96 cell culture microplate was precoated with poly‐L‐lysine (P4832, Sigma Aldrich) 1 day before performing the experiment. BMMC were seeded at a density of 1 × 10^5^ cells/well in 180 μL of XF medium (RPMI medium supplemented with 25 mM glucose, 1 mM pyruvate and 2 mM glutamine). Basal oxygen consumption rate (OCR) and extracellular acidification rates (ECAR) were measured by sequential addition of 1 μM oligomycin (O4876, Sigma Aldrich), 1.5 μM carbonyl cyanide 4‐(trifluoromethoxy) phenylhydrazone (FCCP; C2920, Sigma Aldrich), 0.5 μM of rotenone and antimycin A (557,368 and A8674, both from Sigma Aldrich) and 50 mM 2‐deoxyglucose (2‐DG; D8375, Sigma Aldrich). Metabolic parameters were evaluated based on the manufacturer's instructions (Agilent Technologies).

### 2.8 Senescence‐Associated β‐Galactosidase (SA‐β‐Gal) Activity

One million cells were resuspended in 200 μL of PBS before being placed onto 12 mm circular coverslips contained in 24‐well plates and left 30 min at RT. Then, PBS was removed and the cells were incubated for 20 min with the fixing solution provided in the Cellular Senescence Assay Kit (KAA002, Merck‐Millipore). Slides were then washed twice with 500 μL PBS and incubated with X‐Gal solution (20 mg/mL, Gold BioTechnology, X4281C) for 4 h at 37°C without CO_2_ and protected from the light following the manufacturer's instructions. Then, BMMC were washed twice with PBS and mounted on slides using polyvinyl alcohol before imaging in a LEICA ICC50 E microscope using LAS EZ 3.2.0 software. In parallel assays, enzyme activity was determined by washing the cells twice with PBS and lysing them in 100 μL of a 0.5% Triton X‐100 solution. X‐Gal substrate (20 mg/mL) was added, and tubes were incubated at 37°C for 4 h. Optical density was quantified at 590 nm using a Tecan Sunrise plate reader and the Magellan 7.3 software.

### 2.9 RNA Isolation and RT‐PCR

Total RNA from non‐senescent or senescent BMMC was isolated using TRI Reagent (T9424, Sigma‐Aldrich) and 1 μg was converted to cDNA with a cDNA Synthesis kit (K1622, Thermo). Semiquantitative PCR amplification conditions were as follows: hot start at 94°C for 5 min, denaturation at 94°C for 1 min, annealing at 60°C for 2 min and 30 s, and extension at 72°C for 10 min. The optimal number of cycles was experimentally determined for each amplified gene, and it was found that 25 cycles were within the linear range of the amplification curve as described (Gonzalez‐Espinosa et al. 2003; Medina‐Tamayo et al. 2011). PCR products were separated by 2% agarose gel electrophoresis and visualized by ethidium bromide staining. A 100‐bp DNA ladder was used as a molecular weight marker. Gels were photographed using the MiniBIS Pro System DNR Bio‐Imaging Systems, with the Gel Quant Express software. Utilized primers for semiquantitative PCR are described in Table 1. When stated, quantitative assays were conducted using RealQ Plus (Ampliqon, A323402). The reaction mix was composed according to the manufacturer's protocol using a Qiagen Rotor Gene Q Real‐Time System with the following cycling conditions: 95°C for 2 min, 40 cycles at 95°C for 5 s and 60°C for 30 s. Two replicates were conducted for each data set. Relative expression and the comparative cycle threshold (CT) method (2^−∆∆CT^) were used to calculate relative gene expression under experimental and control conditions normalized to *Gapdh*. Primers for qRT‐PCR are described in Table 1.

**TABLE 1:** Primers used for RT‐PCR and RT‐qPCR.

RT‐PCR			
Gene	Oligonuecleotides	Amplicon size	References
*Il‐1α*	Fw: CTCTAGAGCACCATGCTACAGAC Rw: TGGAATCCAGGGGAAACACTG	310 bp	(Nakasaki et al. 2015)
*Il‐1β*	Fw: TGGACCTTCCAGGATGAGGACA Rw: GTTCATCTCGGAGCCTGTAGTG	148 bp	
*Cxcl‐1*	Fw: ACCCGCTCGCTTCTCTGT Rw: CACCTTTTAGCATCTTTTGG	268 bp	(Zou et al. 2016)
*Il‐6*	Fw: ATGAAGTTCCTCTCTGCAAGAGACT Rw: CACTAGGTTGCCGAGTAGATCTC	640 bp	(Chang et al. 2000)
*Tnfa*	Fw: TTCTGTCTACTGAACTTCGGGGTGATCGGTCC Rw: GTATGAGATAGCAAATCGGCTGACGGTGTGGG	354 bp	
*Gapdh*	Fw: TGAAGGTCGGTGTGAACGGATTTGGC Rw: CATGTAGGCCATGAGGTCCACCAC	980 bp	
*Tgf‐β*	Fw: CGCAACAACGCCATCTATGAGAAA Rw: TTGCAGGAGCGCACAATCATGTTG	818 bp	(Ramírez‐Moreno et al. 2020)
*Ccl‐2*	Fw: ACTCAAGCCAGCTCTCTCTT Rw: TTCCTTCTTGGGGTCAGCAC	274 bp	
*Il‐10*	Fw: ATGCAGGACTTTAAGGGTTACTTGGGTT Rw: ATTTCGGAGAGAGGTACAAACGAGGTTT	455 bp	(Adebanjo et al. 1998)
*Vegf*	Fw: ACATCTTCAAGCCGTCCTGTGT Rw: AAATGGCGAATCCAGTCCCACG	484, 616, 688 bp	(Matsuura et al. 2002)

RT‐qPCR			
*Gapdh*	Fw: ATTGTGGAAGGGCTCATGAC Rw: AGTGGATGCAGGGATGATGT	126 bp	Primer bank (https://pga.mgh.harvard.edu/primerbank/)
*Cxcl‐1*	Fw: AGCTGCGCTGTCAGTGCC Rw: CAAGCCTCGCGACCATTC	136 bp	
*Il‐1 β*	Fw: TGGACCTTCCAGGATGAGGACA Rw: GTTCATCTCGGAGCCTGTAGTG	148 bp	
*Il‐6*	Fw: TACCACTTCACAAGTCGGAGGC Rw: CTGCAAGTGCATCATCGTTGTTC	116 bp	
*p21*	Fw: CGAGAACGGTGGAACTTTGAC Rw: CAGGGCTCAGGTAGACCTTG	106 bp	(D’Angelo et al. 2018)
*p16*	Fw: CCCAACGCCCCGAACT Rw: GCAGAAGAGCTGCTACGTGAA	79 bp	(Edwards et al. 2007)

### 2.10 Degranulation Assays/β‐Hexosaminidase Release

Two million cells were stimulated with distinct amounts of the antigen (Ag) 2–4‐dinitrophenyl coupled to human serum albumin (DNP‐HSA) for 30 min at 37°C in 1 mL of Tyrode's/BSA. After this treatment, cells were placed on ice for 2 min and centrifuged at 12,000 × g for 10 min at 4°C. Sixty microliters of supernatant or triton‐treated cell pellet were placed per well in a 96‐well plate containing 40 μL of 1 mM p‐nitrophenyl‐N‐acetyl‐β‐D‐glucosaminide (PNAG), and incubated for 1 h at 37°C before the addition of 120 μL of stop solution (Na_2_CO_3_ 0.1 M/Na_2_HCO_3_ 0.1 M). Activity of β‐Hexosaminidase (β‐Hex) was quantified by spectrophotometry in an ELISA plate reader Tecan Sunrise at 405 nm, using the Magellan 7.3 software as described (Saitoh et al. 2000).

### 2.11 Intracellular Calcium Determination

Eight million cells were washed once and suspended in Tyrode's/BSA buffer in the presence of 5 μM Fura 2‐AM (F1201, Thermo Fisher Scientific) for 30 min at 37°C. Then, cells were placed in a 2 mL cuvette and introduced in a Fluoromax 3 spectrofluorometer (Jobin Yvon). Basal fluorescence was recorded during 200 s before the addition of 27 ng/mL of DNP‐HSA. *i*[Ca2^+^] was calculated using parameters and the equation previously described (Grynkiewicz et al. 1985).

### 2.12 Western Blot

Two million BMMCs were stimulated and lysed in 200 μL of 2X Laemmli buffer. Fifty μL and 20–50 μg protein were loaded and separated by 12%–16% polyacrylamide gels and transferred to PVDF membranes (Merck, IPVH00010). Blots were incubated in the blotting grade blocker (Bio‐Rad, 1706404) for 1–2 h at room temperature with shaking. Blots were then incubated with primary antibody overnight at 4°C with gentle shaking. Primary antibodies were used in TBS‐T (Tris 50 mM, pH 7.5, NaCl 150 mM, Tween‐20 0.1%) at the following dilutions: p‐ERK 1/2 1:15,000; p‐p65 NFκB 1:5000, p‐Src Tyr416 1:5000, p38 1:5000, p16 1:2000, p21 1:3000, Actin 1:5000. Blots were washed at least three times for 10 min before incubation with appropriate HRP‐conjugated secondary antibodies (diluted 1:10,000) at RT for 1 h with shaking. After 3–4 washes, Immobilon HRP substrate (Millipore, WBKLS0500) was applied. Some of the images were obtained by Medical x‐ray Blue (Carestream, 6041222) and captured by DNR MiniBIS Pro. Densitometric analysis of those blots was performed with the GelQuant Express software. Other western blot images were obtained and quantified with a BioRad Chemidoc XRS Universal Hood, Serial Number IIC721BR09405 and included software.

### 2.13 Confocal Microscopy

One million cells were placed on electrocharged slides for 20 min to allow adhesion in a volume of 300 μL of RPMI medium. Afterward, the medium was removed, and three washes were performed with 1X PBS. Then, blocking was carried out with 300 μL of blocking solution (1X PBS, 0.3% BSA, 5% donkey serum, and 0.001% Tween‐20) for 2 h at RT. Incubation with anti‐p16 (1:200) and anti‐p21 (1:250) antibodies was performed overnight at 4°C in a humid chamber. The next day, slides were washed 5 times with 1X PBS and incubated with the secondary antibody goat anti‐mouse Alexa 488 (1:500, Invitrogen, A11001) for 2 h at room temperature. Then, slides were washed 5 times with 1X PBS and incubated with DAPI (1:500) for 5 min, followed by two additional washes with 1X PBS. Finally, the slides were sealed with DABCO (Sigma‐Aldrich) solution. Images were acquired with a 63× objective using a 4× optical zoom on a DMi8 Stellaris 5 confocal microscope, utilizing Leica Application Suite (LAS) X software, version 4.3.0.24308.

### 2.14 Cytokine Secretion

Quantification of secreted cytokines and chemokines was carried out using a LEGENDPlex mouse inflammation panel (740446) and mouse proinflammatory chemokine panel (740007) according to the manufacturer's recommendations (BioLegend, San Diego, CA, USA). Briefly, a mixture containing 25 μL of assay buffer plus 25 μL of sample plus 25 μL of premixed beads was added to one well of a 96‐well V‐bottom plate and incubated at RT for 2 h with constant shaking and protected from light. Afterwards, the plate was washed with 200 μL of wash buffer 1× into each well and centrifuged at 200× g for 5 min. The supernatant was removed, 25 μL of detection antibody was added to each well and incubated for 1 h at RT/shaking/protected from the light. Then, 25 μL of SA‐PE was added to each well directly and incubated for another 30 min. The plate was washed, and samples were resuspended in 150 μL of wash buffer. Fluorescence intensities to the multiple analytes were recorded using a NovoCyte Quanteon (Agilent) flow cytometer. Data were analyzed with the specific software provided by Biolegend (San Diego, CA, USA). A standard curve was performed in each experiment made according to the manufacturer's instructions.

When necessary, TNF, IL‐6, CCL2, and VEGF secretion was measured using PeproTech ELISA kits (see previous sections) following the manufacturer's instructions.

For intracellular TNF detection, peritoneal cells obtained from the peritoneal lavages were incubated for 3.5 h with vehicle or 100 ng/mL LPS (L8274 Sigma Aldrich, Saint Louis, MO, USA) and GolgiPlug (1:1000; BD Bioscience, San José, CA, USA) in culture media at 37°C. Subsequently, Ghost Dye stain Red 780 was added to exclude dead cells. Surface staining was performed using PE‐Cy7 anti‐CD45, APC anti‐FcεRI, VB785 anti‐c‐kit, and cells were permeabilized and fixed using Cytofix/Cytoperm kit (BD Biosciences, San Jose, CA, USA) according to the manufacturer's instructions. Then, the intracellular TNF was stained with BV650 anti‐TNFα (563943 BD Biosciences). Data were collected using NovoCyte Quanteon flow cytometer (Agilent) and analyzed with the FlowJo software (v10.8). The gating strategy used is described in Figure S7.

### 2.15 Chemotaxis Assays

Cells were washed with 1X PBS and incubated with 1 μg/mL calcein‐AM (Sigma‐Aldrich, C1359) at 37°C for 1 h. Then, cells were washed twice with 1X PBS and resuspended in migration medium (MM; serum‐free RPMI‐1640, pH 7.4). A 48‐well Boyden chamber (Neuro Probe) was employed for the migration assays. Polycarbonate filters (25 × 80 mm) with an 8 μm pore size were coated with 2% bovine skin gelatin (Sigma‐Aldrich) for 2 h at 37°C and then were air‐dried. In the bottom wells of the chamber, 30 μL of distinct media were added: migration medium (MM) as a negative control, sphingosine 1‐phosphate (S1P, 100 nM, Sigma‐Aldrich, S9666) and LPS (10 ng/mL). In each upper well, 40,000 BMMC were placed. The chamber was then incubated for 3 h in a humidified incubator at 37°C with 5% CO₂. After the incubation, migrated cells adhering to the gelatin‐coated filters were visualized using a 20× objective with a 1× optical zoom on a DMi8 Stellaris 5 confocal microscope. Quantification was carried out considering four different fields randomly selected in each preparation using ImageJ software (Java, 2019).

### 2.16 Reconstitution of c‐*Kit* ^*Wsh/Wsh*^ Mice

MC‐deficient mice c‐*Kit* ^*Wsh/Wsh*^ (8–12 weeks old) were reconstituted by an intravenous injection with 2X10^6^ BMMC from 5 weeks old cultures generated as described previously. In all adoptive transfer assays, cells were concentrated in a total volume of 200 μL of sterile BSA‐free Tyrode's buffer and were injected in the tail stem vein of mice, as described (Grimbaldeston et al. 2005; Piliponsky et al. 2010). Eight weeks after injection of BMMC, mice were used for experiments. For long‐term reconstitution, 8–12 weeks old mice were administered with BMMC by the tail vein and maintained in adequate housing conditions until mice were 60 weeks old.

### 2.17 Mouse Endotoxemia Model and Peritoneal Lavages

Mice aged 8–12 weeks or 55–60 weeks were challenged intraperitoneally with LPS (1 mg/kg) or the same volume of vehicle (commercial physiologic saline solution). One hour later, animals were euthanized and peritoneal lavage was performed using a sterile 3 mL syringe containing 2 mL of isotonic saline solution. Approximately 1 mL of lavage was recovered as described previously (Madera‐Salcedo et al. 2011). Peritoneal washes were centrifuged at 3750 × g for 5 min at 4°C, and the supernatants were stored at −80°C until further analysis.

### 2.18 Statistical Analysis

Statistical analyses were made using GraphPad Prism v.9.5.0 (GraphPad Software LLC). For comparison between two groups, results were analyzed using a two‐tailed paired *t* test. For comparison among groups, results were analyzed using one‐way ANOVA, Tukey's multiple comparisons test, and for comparison between multiple groups, two‐way ANOVA with Tukey's or Bonferroni's multiple comparisons test was used. A *p‐*value lower than 0.05 was considered statistically significant. Data are represented as the mean ± SEM unless otherwise noted in the figure legend.

## 3 Results

### 3.1 Long‐Term Culture of BMMC Promotes the Expression of Main Senescence Markers

**FIGURE 1:**
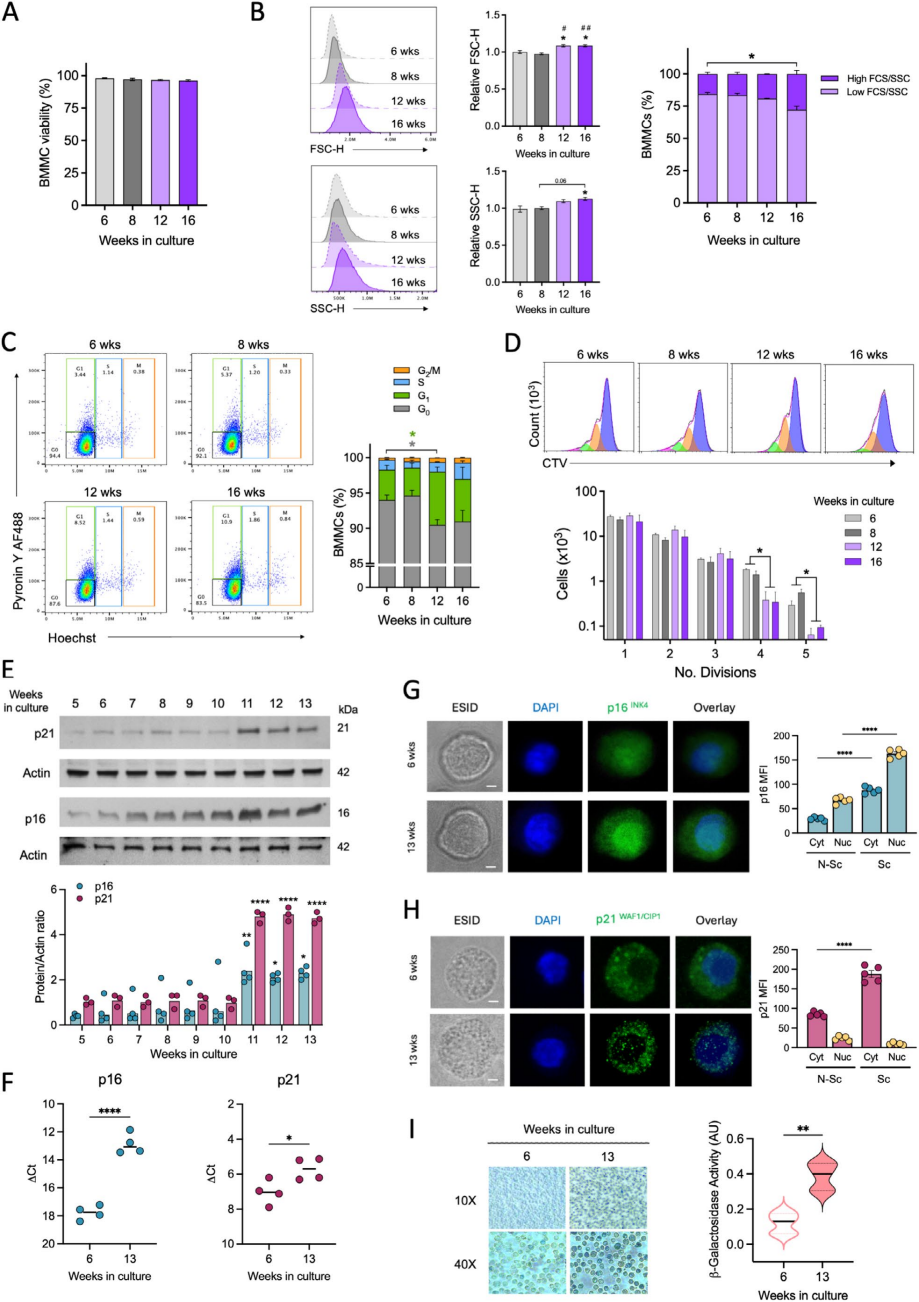
Long‐term culture of BMMC promotes the expression of senescence markers. (A) Cell viability of BMMC cultured during distinct times, determined by flow cytometry. (B) Left and central panels. Cell size and granularity of BMMC cultured for distinct times; one‐way ANOVA, Tukey's multiple comparisons test, *p ≤ 0.05 versus 6 weeks; #*p* ≤ 0.05 and ##*p* ≤ 0.001 versus 8 weeks, *n* = 3–4. Right panel. Proportion of cells with high or low FCS/SCS in BMMC cultures calculated by flow cytometry. Two‐way ANOVA, Tukey's multiple comparisons test, *p ≤ 0.05; *n* = 3–4. (C) Cell cycle analysis in BMMC cultures performed utilizing Pyronin Y and Hoechst positivity. Representative dot plots are shown in left images, whereas quantification of independent experiments is shown in right panel. Two‐way ANOVA, Tukey's multiple comparisons test, *p ≤ 0.05, *n* = 3–4. The color of the asterisk (*) indicates the cell cycle phase being compared. (D) BMMC maintained in culture for distinct times were labeled with CellTrace Violet dye and placed in culture for 7 days. Cell proliferation was evaluated by flow cytometry. Histograms show representative data, and the bars graph shows the cells that went into division in each peak. One‐way ANOVA, Tukey's multiple comparisons test; *p ≤ 0.05; *n* = 2–3. (E) BMMC were cultured over several weeks and the expression of p21 and p16 was assessed by western blot. Upper panel show a representative blot, and lower panel shows the quantification of three independent experiments. One‐way ANOVA, Tukey's multiple comparisons test; *p ≤ 0.05, **p ≤ 0.01, ****p ≤ 0.0001; *n* = 3–4. (F) BMMC were cultured 6 or 13 weeks and total RNA was extracted to perform RT‐qPCR. ΔCt values of p16 and p21 expression are shown. *t*‐test, *p ≤ 0.05, ****p ≤ 0.0001, *n* = 4. (G) Representative confocal images of p16 expression (right panel) and cumulative mean fluorescence intensity (MFI) for cytoplasmic (Cyt) and nuclear (Nuc) localization (left panel) of that protein in BMMC cultured for 6 weeks (non‐senescent, N‐Sc) and 13 weeks (senescent, Sc). ESID (Extended Signal Intensity Detection) photograph, nuclei (blue) and p16 (green). Scale bar = 5 μm. Two‐way ANOVA, Bonferroni's multiple comparisons test; ****p ≤ 0.0001; *n* = 5. (H) Representative confocal images of p21 expression (right panel) and mean fluorescence intensity (MFI, right panel) for cytoplasmic (Cyt) and nuclear (Nuc) localization of that protein in BMMC cultured for 6 weeks (non‐senescent, N‐Sc) and 13 weeks (senescent, Sc). ESID photograph, nuclei (blue) and p21 (green). Scale bar = 5 μm. Two‐way ANOVA, Bonferroni's multiple comparisons test. ****p ≤ 0.0001; *n* = 5. (I) Determination of SA‐β‐Gal activity in BMMC cultured for 6 weeks or 13 weeks. Left panel, representative images of SA‐β‐Gal expression in BMMC at 10× and 40× magnification. Right panel, cumulative of SAβ‐Gal activity; Mann–Whitney test, **p ≤ 0.007. *n* = 5. AU, arbitrary units.

To investigate the changes that replicative senescence induces in MCs, BMMC were differentiated using standardized conditions to obtain cultures showing size and granularity consistent with MC, together with high expression of FcεRI receptor (Meurer et al. 2016). As widely reported, after five or 6 weeks of culture in the presence of IL‐3, cultures with more than 95% of FcεRI^+^, c‐Kit^+^ BMMC were obtained. Since before this time in culture BMMCs show an immature phenotype characterized by a lack of granules in the cytosol (Dvorak 2005), and around 5 to 6 weeks, cells present morphological and functional characteristics of mature mast cells, 5‐to 6‐week‐old cultures were considered the starting mature MC preparation. Then, cultures were maintained up to 16 weeks, changing media once a week as described in the Material and Methods section, and distinct parameters such as cell viability, cell size, and membrane FcεRI receptor expression were analyzed by flow cytometry (general gating strategy is depicted in Figure S1). Long‐term (16 weeks) BMMC culture did not cause significant changes in cell viability (Figure 1A), but it caused an increase in the number of cells showing high forward scattering and side scattering (FSC‐H and SSC‐H) (Figure 1B), suggesting increased size and granularity. When the cell cycle was analyzed (Figure 1C), a significant increase in the number of cells in G_1_ (green part of the bars) accompanied with a decrease in the number of cells in G_0_ (gray part of the bars) was observed at 12 and 16 weeks of culture. To determine the replicative capacity of BMMC, cells from cultures of different ages were loaded with Cell‐Trace Violet (CTV) and cultured for 8 days to monitor the dye dilution upon basal conditions. BMMC from cultures of 6 and 8 weeks old exhibited active cell division, with cells undergoing 1 to 5 divisions in the period tested. However, cells from cultures of 12 and 16 weeks reached only 1 to 3 division cycles during the same time, showing an important decrease in the proportion of cells that reached 4–5 divisions (Figure 1D) and suggesting a blockage in cell cycle progression.

Expression of the negative regulators of cell division p16 and p21 was assessed in BMMC cultures from 5 to 13 weeks. Utilizing western blot, it was observed that p21 and p16 expression levels were low in cultures from 5 to 10 weeks but significantly increased in cultures from 11 to 13 weeks (Figure 1E). Changes in the expression of p21 and p16 were also detected by RT‐qPCR, and it was observed that in 13‐week‐old‐cultures, both mRNAs were increased (Figure 1F). Since it has been reported that the main activity of p16 and p21 occurs in the nuclei but can also occur in the cytoplasm, we analyzed the subcellular localization of those molecules in short‐term (6 week‐old) or long‐term (13 week‐old) BMMC cultures by confocal microscopy. It is of note that, when observed under the microscope, autofluorescence of non‐stained BMMCs was higher in cells from 13‐week‐old cultures compared with those from 6‐week‐old cultures (Figure S2). In cells from long‐term cultures, an increase in total p16 fluorescence was detected in comparison with cells from short‐term cultures (Figure 1G). In 13‐week‐old cultures, p16 was mainly located in the nucleus (Nuc), although cytoplasmic (Cyt) expression was also augmented. On the other hand, p21 was detected in the cytoplasm and in the nucleus of cells from short‐term cultures (6‐week‐old) and its expression was significantly increased in cells from long‐term cultures (13‐week‐old) (Figure 1H).

Activity of SA‐βGal was determined in cells from short‐term and long‐term cultures. As observed in Figure 1I, although BMMC from short‐term cultures expressed detectable amounts of β‐Gal, BMMC from long‐term cultures showed a significant increase in the activity of that enzyme.

### 3.2 Senescent BMMC Express a Complex SASP and Altered Metabolic Parameters

**FIGURE 2:**
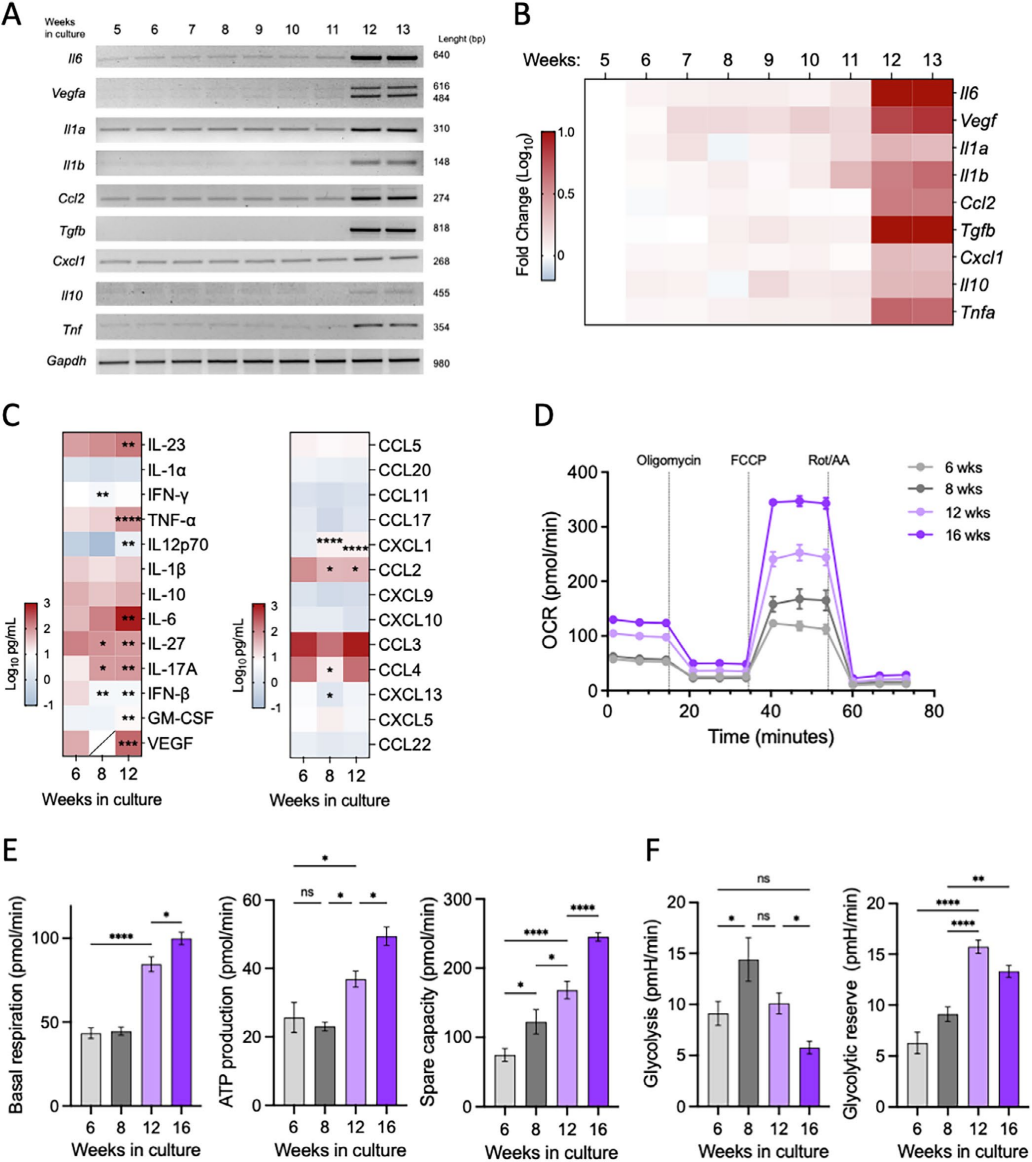
Long‐term culture of BMMC lead to the expression of senescence‐associated secretory phenotype (SASP) and altered metabolism. (A) Semi‐quantitative RT‐PCR analysis of cytokine expression from BMMC cultures with different ages. A representative gel from three independent experiments is shown. (B) Quantification of cytokine mRNA expression from data shown in panel A, represented as fold change relative to the expression levels at 5 weeks of culture. (C) Cell supernatants from cultures with different ages were analyzed for detection of distinct cytokines by LEGENDPlex assays. Data represent Log_10_ of pg/mL of each evaluated cytokine. Diagonal line means that the cytokine was not determined. One way ANOVA, Tukey's multiple comparisons test. *p ≤ 0.05, **p ≤ 0.01, ***p ≤ 0.005, ****p ≤ 0.001. (D) Oxygen Consumption Rate (OCR) obtained from two independent experiments is shown. Dashed line indicates the addition of specific compounds into the media that is, oligomycin, carbonyl cyanite‐4 (trifluoromethoxy) phenylhydrazone (FCCP), and Rotenone/Antimycin A (Rot/AA). (E) Comparisons of mitochondrial respiratory parameters of resting BMMC, basal OCR, ATP production and spare capacity. (F) Comparisons of glycolytic parameters, glycolysis and glycolytic reserve. Data are mean ± SEM of the three measurements of four independent cell plating seahorse microplate wells. One way ANOVA, Tukey's multiple comparisons test. *p ≤ 0.05, **p ≤ 0.01, ****p ≤ 0.0001; *n* = 3–4. ns, not significant.

Acquisition of SASP was evaluated in long‐term BMMC cultures. First, RT‐PCR was performed to determine the amounts of distinct cytokine mRNAs in cells from cultures with different ages. In comparison with cells from 5‐and 6‐week‐old cultures, those from 12 and 13‐week‐old cultures showed increased basal levels of *Il6, Vegf, Il1a, Il1b, Ccl2, Tgfb, Cxcl1, Il10*, and *Tnf* mRNAs (Figure 2A,B). To analyze the secretion of cytokines and chemokines, a LegendPlex flow cytometry kit was utilized. Increased secretion of IL‐23, TNF, IL‐6, VEGF, GM‐CSF, IL‐17A, and CXCL1 was detected in cells from 12‐week‐old cultures in comparison with cells from 6‐week‐old cultures. Also, a decrease in IL‐27, IFN‐β, CXCL10, and CCL2 production was observed in 12‐week‐old cultures (Figure 2C and Figure S3).

Key parameters of mitochondrial respiration were then assessed. First, we evaluated the metabolic profile using a flux analyzer to monitor the mitochondrial oxygen consumption rate (OCR) and the extracellular acidification rate (ECAR) in MCs with different weeks of culture (Figure 2D). As observed, respiratory capacity was found to be increased in cells from 12 and 16‐week‐old cultures. In addition, OCR‐related parameters: basal respiration, ATP production, and spare capacity were higher in BMMC from 12 and 16‐week‐old cultures (Figure 2E). ECAR evaluation showed that BMMC from 12 and 16‐week‐old cultures displayed lower basal glycolysis and higher glycolytic reserve compared to those from 6‐and 8‐week‐old cultures (Figure 2F).

Due to their morphological changes, expression of SA‐β‐Gal, expression of p16 and p21, increased cytokine secretion, and altered metabolic parameters, BMMC from 12 to 16 weeks old cultured were considered senescent cells (Sc), whereas cells from 6 to 8 weeks old cultures were considered non‐senescent (N‐Sc) ones.

### 3.3 Senescence Increases FcεRI‐Induced Responses in BMMC

**FIGURE 3:**
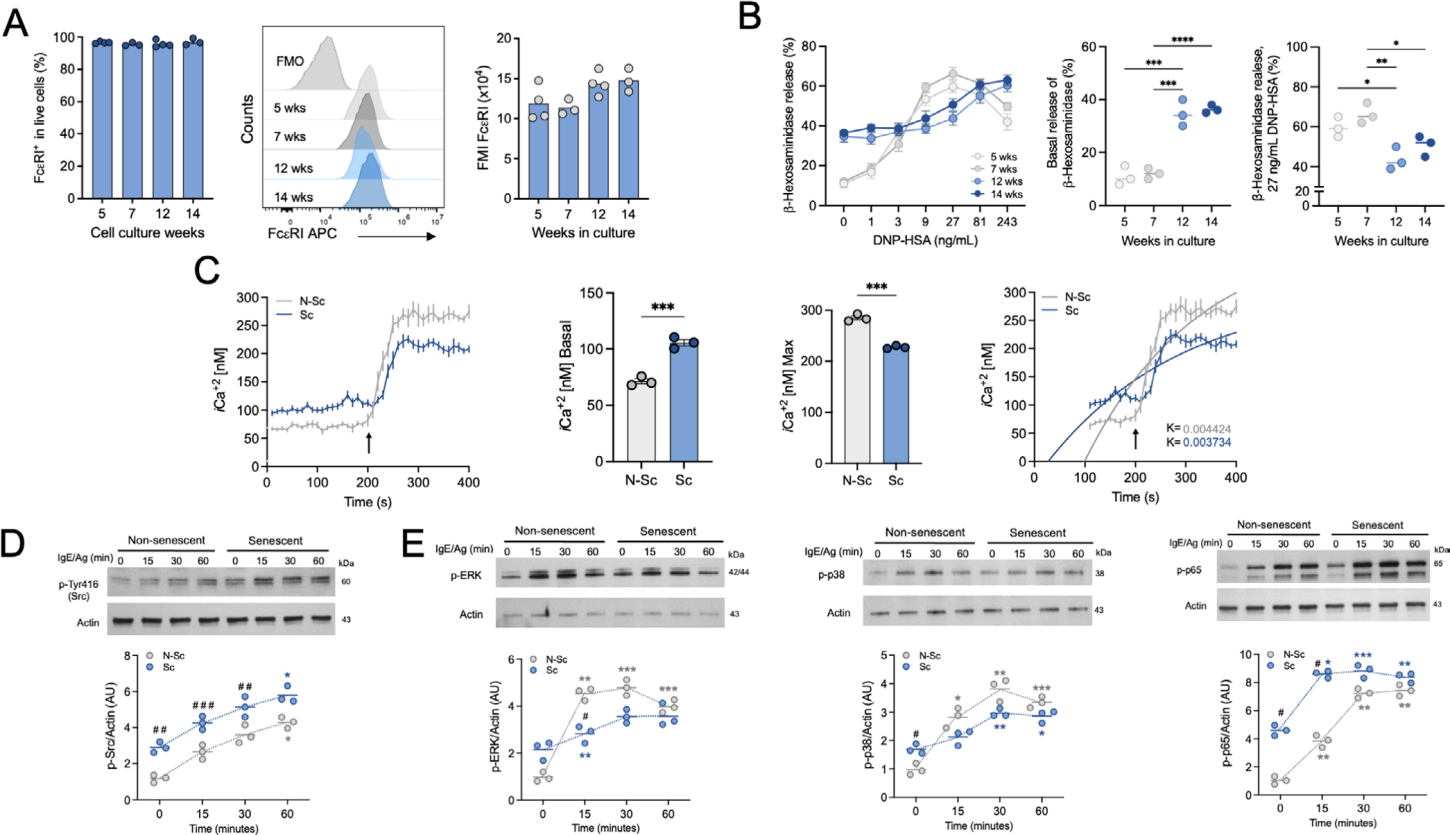
Senescence modifies FcεRI‐dependent responses in BMMC. (A) Expression of FcεRI was determined by flow cytometry in BMMC cultures of distinct ages. *n* = 3–4. (B) Dose–response curve of β‐Hexosaminidase release in response to antigen. Dot plots presented in left panel show the response induced by different concentrations of antigen (mean ± SEM), being middle panel basal levels and right panel maximal levels at 27 ng/mL antigen concentration. Two‐way ANOVA, Bonferroni's test, *p ≤ 0.05, **p ≤ 0.01, ***p ≤ 0.001, ****p ≤ 0.0001; *n* = 3. (C) Changes in intracellular calcium concentration in response to antigen addition was determined in senescent and non‐senescent MC. First panel shows changes in *i*[Ca^2+^] concentration after antigen addition. Basal values were recorded for 200 s and then cells were stimulated with 27 ng/mL Ag (black arrow). Measurement was followed by 400 s after Ag addition. Second and third panels show basal and maximal *i*[Ca^2+^] values of non‐senescent (N‐Sc) and senescent (Sc) BMMC. Unpaired t test, ***p ≤ 0.001. Fourth panel shows the rate constant (K), calculated by fitting a mono‐exponential function to calcium mobilization data, *n* = 3. (D and E) Time course of protein phosphorylation following FcεRI receptor activation. Total protein extracts were obtained at specified time points after antigen addition (27 ng/mL) and p‐Src (Y416), p44/p42 ERK, p‐38 and p65NFκB were detected by western blot. Two‐way ANOVA, Bonferroni's comparisons test, *p ≤ 0.05, **p ≤ 0.005, ***p ≤ 0.001 versus baseline (0 min); #*p* ≤ 0.05, ##*p* ≤ 0.005, ###*p* ≤ 0.0005 versus N‐Sc, *n* = 3.

Effector activities of senescent MCs were analyzed by triggering the FcεRI receptor. More than 95% of live senescent cells expressed that receptor in their plasma membrane and, although the fluorescence mean showed a tendency to augment with culture age, this change was not statistically significant (Figure 3A). To assess the degranulation response, IgE‐sensitized cells were stimulated with the specific antigen DNP‐HSA, and the activity of β‐Hexosaminidase (β‐Hex) in the supernatant was determined (Figure 3B). Non‐senescent BMMC secreted close to 10% ± 3% of their total β‐Hex content in basal (non‐stimulated) conditions, and this value increased to 70% ± 7% in response to antigen (27 ng/mL). In senescent BMMC, basal secretion of β‐Hex increased to 34% ± 3%, whereas the maximal secretion was 60% ± 4% and was obtained with higher concentrations of antigen (81 ng/mL). At 27 ng/mL of antigen, senescent cells secreted less β‐Hex than non‐senescent BMMC (50% ± 3% and 66% ± 3%, respectively). Since degranulation requires an increase in intracellular calcium concentration, we decided to analyze possible alterations in calcium mobilization in senescent BMMC. The first panel of Figure 3C shows a representative kinetics of calcium mobilization after Ag addition (27 ng/mL, black arrow) to non‐senescent and senescent BMMC. Basal (_*i*_[Ca^2+^]) was higher in senescent BMMC, but maximal intracellular calcium concentrations after antigen addition were reached only in non‐senescent cells. Values of basal _*I*_[Ca^2+^] of senescent cells were close to 40% higher than those observed in non‐senescent BMMC (middle panels of Figure 3C), while the maximal calcium concentration reached after Ag addition was significantly lower. Also, the rate of intracellular calcium increase after Ag addition was higher in non‐senescent cells compared with that observed in senescent cells (last panel of Figure 3C).

Phosphorylation of key proteins downstream the signaling cascade of IgE receptor was analyzed. Basal phosphorylation of the early kinase Src and the MAPKs ERK1/2 and p38, together with the transcription factor NFκB (p65 protein) was higher in senescent BMMC than in non‐senescent ones (Figure 3D,E). Remarkably, after IgE/Ag addition, a lower maximal phosphorylation of those proteins was observed in senescent cells when compared with their respective basal values.

### 3.4 Senescence Alters TLR4‐Induced Cytokine Secretion and Chemotaxis

**FIGURE 4:**
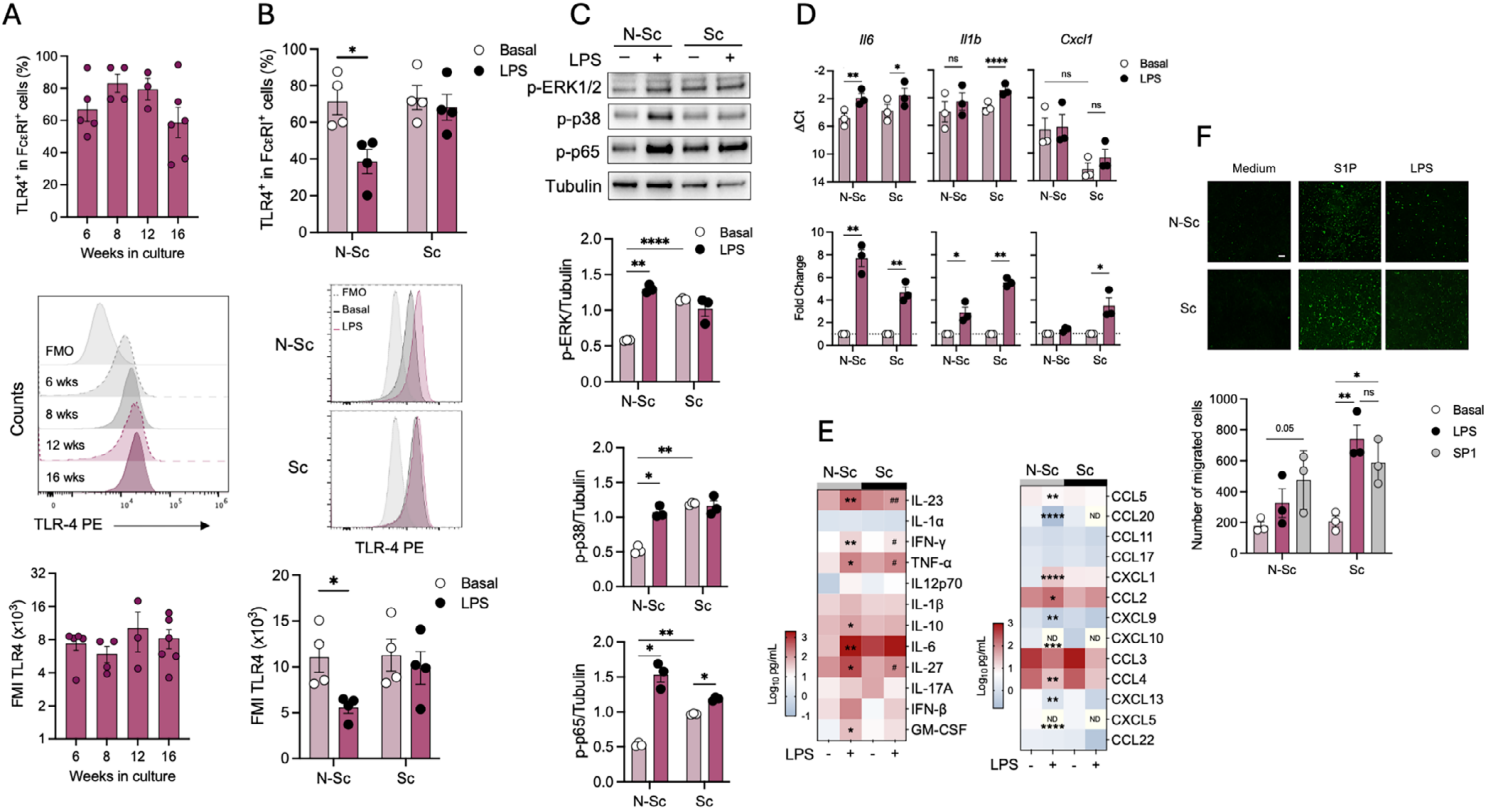
Senescent BMMC show altered responses to TLR4 receptor activation. (A) BMMC from cultures of distinct ages were analyzed for the presence of TLR4 receptor by flow cytometry. Upper panel shows the percentage of FcεRI^+^ cells expressing TLR4 receptor. Middle panel shows representative histograms of TLR4 expression in cultures of distinct ages (6 weeks old, N‐Sc and 13 weeks old, Sc). Lower panel shows FMI values reflecting TLR4 expression in BMMC from distinct cultures. (B) Non‐senescent and senescent BMMC were stimulated with vehicle or LPS (100 ng/mL) for 1 h and then TLR4 presence on plasma membrane was detected by specific anti‐TLR4 antibodies by flow cytometry. Upper panel shows the percentage of TLR4^+^ cells among FcεRI^+^ cells. Middle panel shows representative histograms, and lower panel shows MFI values of TLR4 expression in BMMC cell surface. Two‐way ANOVA, Bonferroni's comparisons test, *p ≤ 0.05, *n* = 3. (C) Non‐senescent and senescent BMMC were stimulated with vehicle or LPS (100 ng/mL) for 30 min and phosphorylation of ERK1/2, p38 and p65 NFκB was determined by Western blot. Upper panel shows a representative experiment, whereas lower panels show quantification of at least three independent experiments. Two‐way ANOVA, Bonferroni's comparisons test, *p ≤ 0.05, **p ≤ 0.05, ***p ≤ 0.001, *n* = 3. (D) Non‐senescent and senescent BMMC were stimulated with vehicle or LPS (100 ng/mL) and total RNA was extracted to perform RT‐qPCR. Upper panel shows ΔCt values and lower panel shows fold change values of *IL6*, *IL1b* and *Cxcl1* mRNAs. Two‐way ANOVA, Bonferroni's comparisons test, *p ≤ 0.05, **p ≤ 0.01, ****p ≤ 0.0001, *n* = 3. ns, not significant. (E) Non‐senescent and senescent BMMC were stimulated with vehicle or LPS (100 ng/mL) and incubated at 37° for 2 h. Cytokine and chemokine concentrations were determined in cell supernatants by LEGENDplex kits. Heat maps represent the concentration in pg/mL (Log_10_) for each evaluated cytokine. Two‐way ANOVA, Tukey's comparisons test *n* = 3, *p ≤ 0.05, **p ≤ 0.01, ***p ≤ 0.005, ****p ≤ 0.001. *Un‐stimulated versus LPS treatment and ^#^N‐Sc versus Sc cells both with LPS. (F) Migration towards sphingosine 1‐phosphate (S1P, 100 nM) and LPS (10 ng/mL) in non‐senescent and senescent BMMC was determined in Boyden's chamber experiments. Two‐way ANOVA, Bonferroni's comparisons test, *p ≤ 0.01, **p ≤ 0.005, *n* = 3. ns, not significant.

BMMC express TLR4 receptor and rapidly respond to acute addition of bacterial lipopolysaccharide (LPS) by synthesizing and releasing distinct cytokines, such as TNF short time after stimulation (Sandig and Bulfone‐Paus 2012; Madera‐Salcedo et al. 2013). In vivo, it has been shown that rapid inflammatory responses depending on TLR4 activation in MC are essential for defense against Gram‐negative bacteria (Echtenacher et al. 1996; Piliponsky et al. 2010; Sandig and Bulfone‐Paus 2012). With this in mind, we sought to determine if senescence could alter the amount of TLR4 receptor and rapid responses to LPS in BMMC. We found that senescence did not significantly modify the number of TLR4^+^ cells among FcεRI^+^ cells in BMMC cultures (Figure 4A). Also, no statistically significant difference was observed in the amount of TLR4 expressed in senescent BMMC compared with non‐senescent cells (middle and lower panels of Figure 4A). Receptor internalization in response to LPS addition was measured in non‐senescent cells and compared with senescent ones. In non‐senescent cultures, after 1 h of LPS (100 ng/mL) addition, the number of cells expressing TLR4 in the plasma membrane diminished by close to 50%, and this was reflected also in FMI values, showing the reported TLR4 internalization after triggering in this cell type (Figure 4B) (Pérez‐Rodríguez et al. 2020). Receptor internalization in response to LPS was not detected in senescent BMMC.

Then, TLR4‐induced early signaling events were determined by WB. LPS addition to non‐senescent cells promoted an important phosphorylation of the MAPKs ERK1/2 and p38 together with the transcription factor p65NFκB (Figure 4C). As previously shown (Figure 3), in comparison with non‐senescent cells, significantly higher levels in basal ERK1/2, p38, and p65NFκB phosphorylation were found in senescent BMMC. Remarkably, after LPS addition, only N‐Sc cells showed a significant increase in protein phosphorylation, and no activation of ERK1/2 and p38 MAPK was observed in Sc cells. Regarding NFκB, only a slight increase in its phosphorylation was found in Sc cells.

To analyze the effect of senescence on cytokine expression after acute TLR4 activation, non‐senescent and senescent BMMC were stimulated with LPS, and selected cytokine mRNAs were analyzed by RT‐qPCR 1 h later. Absolute mRNA levels of *Il6* and *Il1b* and their increase after LPS addition did not change among senescent and non‐senescent cells, whereas *Cxcl1* mRNA levels were lower in senescent cells and did not change in response to LPS (Figure 4D). When fold change in mRNA production triggered by LPS was analyzed, lower levels of *ll6* mRNA were produced in senescent cells, whereas higher amounts of *Il1b* and *Cxcl1* were observed in senescent cells in comparison to non‐senescent ones. Then, cytokine secretion in response to LPS was determined by LEGENDplex, and it was observed that senescence promoted changes in TLR4‐induced cytokine production in BMMC. Compared to basal levels, acute LPS addition (100 ng/mL for 1 h) caused an increase in the secretion of IL‐23, IFNγ, TNF, IL‐10, IL‐6, IL‐27, GM‐CSF, CCL5, CCL20, CXCL1, CCL2, CXCL9, CXCL13 in non‐senescent cells. However, in senescent cells, basal levels of some secreted cytokines were higher (as previously shown) and LPS addition provoked a lower induction compared to those basal values. This was the case for IL‐17A, CCL5, CCL20, CCL3, CCL4, CXCL5 (Figure 4E and Figure S4). Additionally, LPS addition to senescent cells induced higher levels of TNF but lower levels of IL‐23, IFN‐γ and IL‐27. Taken together, these data indicate a reduced sensitivity to TLR4 triggering in senescent MC.

In addition, we determined if senescence could alter the migration of BMMC to the well‐known MC chemoattractant sphingosine 1 phosphate (S1P) and to LPS. A representative migration assay performed in Boyden's chamber is shown in the upper panel of Figure 4F, where increased chemotaxis towards S1P and LPS was observed in senescent cells.

### 3.5 Long‐Term LPS Treatment Promotes Stress‐Induced Senescence of BMMC

**FIGURE 5:**
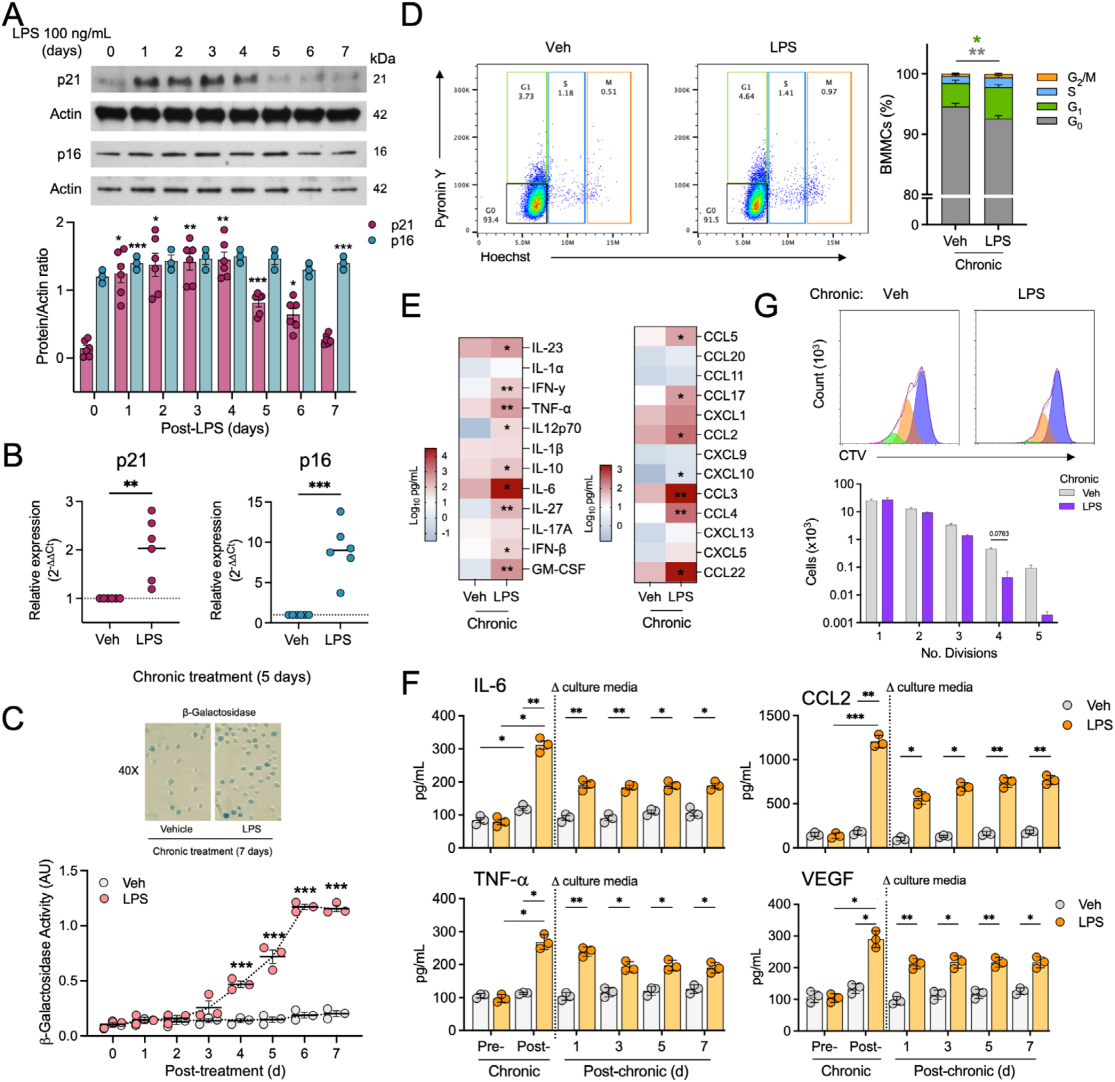
Long‐term presence of LPS causes stress‐induced senescence of BMMC. (A) Top panel shows the expression of the senescence markers p21 and p16 by Western blot, using Actin as a loading control in 6‐week cultured cells exposed to LPS (100 ng/mL) for 1 to 7 days. Bottom panel shows quantification of the results. One‐way ANOVA, Tukey's multiple comparisons test, *p ≤ 0.05, **p ≤ 0.01; ***p ≤ 0.001 versus time 0, *n* = 3. (B) Quantitative RT‐qPCR of the expression of p16 and p21 in cells exposed to vehicle or LPS (100 ng/mL) in a chronic treatment for 5 days. *t*‐test, **p ≤ 0.01, ***p ≤ 0.001, *n* = 6. (C) Top panel shows a representative image of SA‐β‐Gal expression after 7 days of treatment with vehicle or LPS (100 ng/mL). Lower panel exhibits values of SA‐β‐Gal activity from days 1 to 7 of treatment with vehicle or LPS from at least three independent experiments. One‐way ANOVA, Tukey's multiple comparisons test. ***p ≤ 0.001, *n* = 3–4. (D) BMMC were incubated in the presence of LPS (100 ng/mL) for 7 days and the number of cells in G_1_, S and M cell cycle phases was determined by Pyronin Y and Hoechst positivity. Left panel shows a representative experiment and left panel shows results obtained with 3–4 independent cultures. Two‐way ANOVA, Tukey's multiple comparisons test, *p ≤ 0.05, **p ≤ 0.01; *n* = 3–4. The color of the asterisk (*) indicates the phase being compared. (E) Cell supernatants from cultures exposed to vehicle or LPS were analyzed for detection of distinct cytokines by LEGENDPlex. Data represent Log_10_ of pg/mL of each measured cytokine. Paired *t*‐test *n* = 3, *p ≤ 0.05, **p ≤ 0.01. (F) BMMCS were exposed to vehicle or LPS for 5 days and media was collected to determine the content of selected cytokines. After 5 days, cells were washed and resuspended in fresh medium without LPS. Samples of the culture media were then taken 1, 3, 5 and 7 days after the medium change and the amount of IL‐6, CCL2, TNF and VEGF was determined by ELISA. Two‐way ANOVA, Tukey's multiple comparisons test *n* = 3, *p ≤ 0.05, **p ≤ 0.01, ***p ≤ 0.001. (G) BMMC exposed to vehicle or LPS for 5 (100 ng/mL) days were labeled with CellTrace Violet (CTV) dye and placed in culture for 7 days. After that time, cell proliferation was evaluated by flow cytometry. Histograms show representative data, and the bar graph displays the proportion of cells that entered division in each peak. Two‐way ANOVA followed by Tukey's multiple comparisons test, *p* = 0.076; *n* = 3.

To investigate if senescence could be induced by prolonged stress of MCs, we took advantage of previous reports showing that chronic exposure to LPS (for 24 h or more) provokes stress‐induced senescence in distinct cell types (Wang et al. 2020; Wueest et al. 2020; Danish et al. 2025). Non‐senescent BMMC were exposed for several days to LPS (100 ng/mL) and the appearance of senescence markers was determined. Long‐term (7 days) TLR4 stimulation with LPS did not modify cell viability and size or granularity of BMMC (Figure S5). However, chronic treatment with LPS (between 1 and 5 days) induced a transient expression of p21 and a less important, but still significant increase in p16 (Figure 5A). When analysis of mRNA expression of those proteins was performed by RT‐qPCR, a seven‐fold increase in p16 mRNA and a 1.5‐fold increase in p21 mRNA were detected after 5 days of LPS treatment (Figure 5B). At the same time, a gradual increase in SA‐β‐Gal was detected in response to LPS treatment (Figure 5C). Chronic exposure (5 days) to LPS also increased the number of cells in the G_1_ phase of the cell cycle (green section of the bars, Figure 5D) and a reduction in the number of cells in G_0_ (gray section of the bars). When cytokine content in supernatants of chronically LPS‐treated cells was analyzed, we found that LPS treatment for 5 days caused the release of IL‐23, IFNγ, TNF, IL12p70, IL‐10, IL‐6, IL‐27, IFN‐β, GM‐CSF, CCL5, CCL17, CCL2, CXCL10, CCL3, CCL4, and CCL22 (Figure 5E and Figure S6).

Since accumulative LPS‐induced cytokine release (up to 5 days) presented in Figure 5E showed higher values of some cytokines when compared to those obtained with acute treatment (Figure 4E), suggesting continuous secretion of some of them, and cell senescence is related to a permanent change in cell phenotype characterized by the expression of SASP, we decided to investigate whether the chronic treatment with LPS could induce long‐term alterations in cytokine secretion in MC observable even in the absence of LPS. So, cells were exposed to either a vehicle or LPS (100 ng/mL) for 0 or 5 days. Aliquots of culture media were taken and kept frozen at −80°C until use. Then, both groups of cells were washed (to remove LPS) and resuspended in LPS‐free fresh medium at 37°C. Culture media samples were then collected 1, 3, 5, or 7 days after the medium change, and selected cytokines were determined by ELISA (Figure 5F).

As expected, LPS addition for 5 days significantly increased the amount of IL‐6, CCL2, TNF, and VEGF in the medium (Figure 5F, pre‐ vs post‐chronic legend). One day after LPS removal, important amounts of IL‐6, CCL2, TNF, and VEGF were still secreted by BMMCs. However, the amounts of IL‐6, TNF, and VEGF remained constant from 1 day after the change of culture media until day 7, suggesting that, after 1 day, secretion of those cytokines was stopped. Only CCL2 secretion presented the tendency to augment, although values were not statistically significant (Figure 5F, post‐chronic legend).

Finally, the replicative capacity of LPS‐treated cells was determined and is shown in Figure 5G. Prolonged exposure to LPS resulted in an important decrease in the number of cells undergoing 4 to 5 divisions. These data indicate that chronic (5 days) LPS‐induced stress promotes a transient senescent phenotype in BMMC, characterized by increased expression of p16, p21, SA‐β‐Gal activity, cell cycle arrest, reduced cell division, and a characteristic SASP that lasted 24 h in the absence of that ligand.

### 3.6 Aging and Stress‐Dependent Senescence Increase MC‐Dependent TLR4‐Triggered Cytokine Production in Vivo

To investigate if aging could alter MC‐dependent acuteTLR4‐triggered responses in vivo, we took advantage of the well‐characterized model of acute endotoxemia in mice consisting of the intraperitoneal (*i.p*.) administration of LPS and the recovery of peritoneal washes shortly after that inoculation to analyze cells and produced mediators. In that model, it has been widely demonstrated that rapid TNF production after Gram‐negative bacteria or LPS administration relies on MC activation (Malaviya et al. 1996; Echtenacher et al. 1996; Piliponsky et al. 2010; Madera‐Salcedo et al. 2011).

**FIGURE 6:**
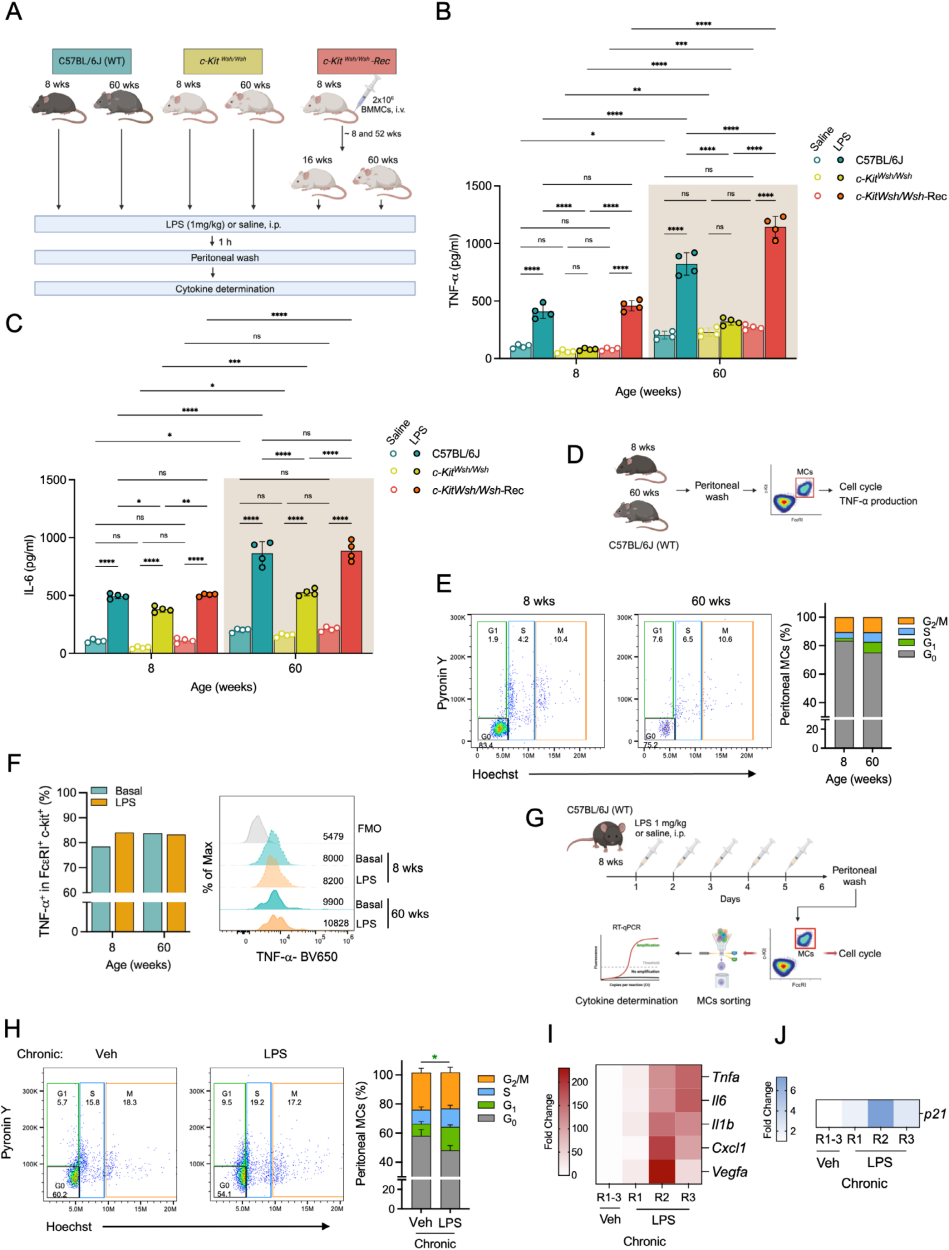
Replicative and stress‐induced senescence promotes hyper‐responsiveness of MC to LPS in vivo. (A) Diagram showing experimental design for endotoxemia assays (see text for details). (B) Vehicle and LPS‐induced TNF production in peritoneal washes from young (8 weeks old) or aged (60 weeks old) C57BL6/J (WT) mice, MC‐deficient *c‐Kit* ^*Wsh/Wsh*^ mice, and *c‐Kit* ^*Wsh/Wsh*^ reconstituted with BMMC derived from WT mice (*c‐Kit* ^*Wsh/Wsh*^‐Rec). (C) Vehicle and LPS‐induced IL‐6 production in peritoneal washes from 8 weeks to 60 weeks old C57BL6/J (WT), *c‐Kit* ^*Wsh/Wsh*^ mice or *c‐Kit* ^*Wsh/Wsh*^ Rec mice. TNF and IL‐6 concentrations were determined by ELISA. Two‐way ANOVA, Tukey's multiple comparisons test, *p ≤ 0.05, **p ≤ 0.01, ***p ≤ 0.001, ****p ≤ 0.0001; *n* = 4; (D) Diagram showing experimental design for isolation of peritoneal MCs from young (8 weeks old) or aged (60 weeks old) mice. (E) Analysis of cell cycle phase in peritoneal MC from young and aged mice. Pool of 4 mice in each age. (F) Basal and LPS‐induced TNF production in peritoneal MC purified from young or aged mice. Pool of 4 mice in each age. (G) Experimental design for stress‐induced senescence in peritoneal cavity. Young WT mice were daily administered *i.p*. with LPS (1 mg/kg) for 5 days before performing peritoneal washes and MC isolation by flow cytometry. (H) Cell cycle analysis of peritoneal MC from animals subjected to multiple administrations of LPS. Two‐way ANOVA, Tukey's multiple comparisons test, *p ≤ 0.05, *n* = 3. Color of the asterisk indicates the phase being compared. (I) Cytokine mRNA production in MC purified from peritoneal cavity of mice repeatedly treated with LPS. R1 to 3 indicate results from three distinct mice subjected to the protocol showed in (G). (J) p21 mRNA production in MC purified from peritoneal cavity of mice repeatedly treated with LPS. R1 to 3 indicate results from three distinct mice as in panel I.

MC‐proficient C57BL6/J (WT), MC‐deficient (*c‐Kit* ^*Wsh/Wsh*^) and *c‐Kit* ^*Wsh/Wsh*^ reconstituted with WT BMMC (*c‐Kit* ^*Wsh/Wsh*^‐Rec) mice of different ages were intraperitoneally administered with saline solution or LPS and, after 1 h, peritoneal washes were performed to determine TNF and IL‐6 production by ELISA (Figure 6A). Absolute values of cytokine content and statistical analysis are presented in Table S1 and Figures 6B,C. In 8‐week‐old WT mice, low basal intraperitoneal levels of TNF (around to 105 ± 8 pg/mL) were observed (Figure 6B). One hour after LPS administration (1 mg/Kg), TNF concentration increased to 409 ± 30 pg/mL. In contrast, in 60‐week‐old mice, basal levels of TNF were higher (213 ± 17 pg/mL) and increased to 822 ± 49 pg/mL after LPS administration. To test if this response was still dependent on the presence of MC in aged animals, MC‐deficient *c‐Kit* ^*Wsh/Wsh*^ of 8 or 60 weeks of age were subjected to the same treatment. As reported, young MC‐deficient mice presented lower levels of intraperitoneal TNF (60 ± 7 pg/mL) and did not produce TNF in response to LPS (detected value = 80 ± 5 pg/mL). However, an increase in basal levels of this cytokine was observed with aging, since 60‐week‐old MC‐deficient mice produced 230 ± 18 pg/mL of TNF, and no additional TNF was produced after LPS administration (detected value = 322 ± 15 pg/mL). As reported, responsiveness to LPS in *c‐Kit* ^*Wsh/Wsh*^ animals was produced when they were reconstituted with BMMC from WT animals. For those experiments, 8‐week‐old mice were intravenously reconstituted with BMMC from WT animals and, 8 or 52 weeks later, saline or LPS were *i.p*. administered and the same procedures described above were followed. In young (16‐week‐old) reconstituted mice, basal levels of TNF in the peritoneal cavity were around 80 ± 7 pg/mL. When LPS was administered, TNF production increased, reaching values close to 459 ± 23 pg/mL. Those values were not statistically different from those observed in young WT mice (Figure 6B). When the response was evaluated in 60‐week‐old *c‐Kit* ^*Wsh/Wsh*^‐Rec mice, an increase in basal levels of TNF was detected (value = 270 ± 11) and, after LPS addition, TNF levels were even higher than those observed in 60‐week‐old WT animals (close to 1143 ± 46 pg/mL).

Additionally, we also determined IL‐6 production in the same peritoneal washes (Figure 6C). In 8‐week‐old WT mice, basal levels of IL‐6 were close to 106 ± 7 ng/mL and, 1 h after LPS addition, those levels reached 494 ± 10 pg/mL. However, in 60‐week‐old WT mice, an increase in basal amounts of IL‐6 were detected (215 ± 8 pg/mL) and hyper‐responsiveness to LPS was observed, since IL‐6 values reached 864 ± 50 pg/mL (Figure 6C). When basal IL‐6 levels were quantified in peritoneal washes of the young MC‐deficient *c‐Kit* ^*Wsh/Wsh*^ mice, those values were not different to the observed in WT mice (49 ± 5 pg/mL). In response to LPS, MC‐deficient mice produced close to 380 ± 12 pg/mL, value that was lower than the observed in young WT mice (Figure 6C). In 60 weeks old MC‐deficient mice, basal levels of intraperitoneal IL‐6 were close to 156 ± 7 pg/mL and this value was not statistically different from the observed in aged WT mice. Addition of LPS to those animals also increased the levels of IL‐6, although values were significantly lower than in aged WT mice (527 ± 15 pg/mL in 60 weeks old *c‐Kit* ^*Wsh/Wsh*^ versus 865 ± 50 pg/mL in 60 weeks old WT mice). When peritoneal washes of 8 weeks old *c‐Kit* ^*Wsh/Wsh*^ *‐Rec* animals were analyzed, basal levels of IL‐6 were close to 110 ± 10 pg/mL, values that were like the observed in young WT mice. After LPS addition, young *c‐Kit* ^*Wsh/Wsh*^ *‐Rec* mice reached 505 ± 6 pg/mL, and that value was not different to the observed in young WT mice but it was significantly higher than the detected in non‐reconstituted young mice (Figure 6C). In 60 weeks old *c‐Kit* ^*Wsh/Wsh*^ *‐Rec mice*, basal values of IL‐6 in peritoneal washes were close to 210 ± 10 pg/mL, value that was not different from the observed in basal 60 weeks old WT mice but higher than the observed in young *c‐Kit* ^*Wsh/Wsh*^ *‐Rec* animals. After LPS addition, IL‐6 values in aged *c‐Kit* ^*Wsh/Wsh*^ *‐Rec* mice reached 885 ± 42 pg/mL, value that was significantly higher than the observed in 6 weeks old *c‐Kit* ^*Wsh/Wsh*^ *‐Rec* in the same condition.

To evaluate if the senescent phenotype of MC could be observed in vivo, peritoneal lavages were performed in both young (8 weeks old) and aged (60 weeks old) WT mice without any treatment. A pool of peritoneal lavages from four mice of each age was made and peritoneal MC (c‐Kit^+^ FcεRI^+^ live cells) were gated utilizing the conditions mentioned in Figure S7. Those cells were analyzed for cell cycle and TNF production by flow cytometry (Figure 6D). Cell cycle phase in gated peritoneal MC was analyzed using Pyronin Y and Hoechst staining (Figure 6E). As observed, fewer MC were recovered from aged animals, and the proportion of MC in G_0_ phase was lower in 60‐week‐old mice. Also, an increase in the number of cells in the G_1_ phase was observed when compared to cells isolated from young animals (Figure 6E).

To directly evaluate the ability of peritoneal MC from aged animals to respond to TLR4 stimulation, FcεRI^+^ c‐Kit^+^ cells from the pool mentioned in Figure 6D were analyzed for TNF intracellular content in basal conditions or after the treatment with LPS (100 ng/mL) for 4 h. The percentage of TNF‐positive MCs was higher in peritoneal cells from aged animals compared to those from young mice. After LPS stimulation, the number of TNF‐positive cells increased among MC gated from young animals, whereas this was not observed in cells from aged animals (Figure 6F). When the mean fluorescence intensity (FMI) of TNF in peritoneal MC was analyzed, the cells gated from aged mice exhibited higher MFI than those from young mice, even after LPS stimulation (Figure 6F).

Finally, to analyze if LPS‐induced stress could lead to senescence of MC in vivo, young WT mice were *i.p*. repeatedly administered with vehicle (saline solution) or LPS (1 mg/Kg) for 5 days. After this, peritoneal washes were performed, and MC were detected (c‐Kit^+^ FcεRI^+^ live cells) and sorted by flow cytometry (Figure 6G) following the strategy shown in Figure S7. MC were then analyzed to detect specific phases of the cell cycle, and results are shown in Figure 6H. Peritoneal MC isolated from LPS‐repeatedly treated animals showed a significant increase in cells in the G_1_ phase of the cell cycle (green section of bars) and a slight reduction in the number of cells in G_0_ (gray section of bars) (Figure 6H). Total RNA was obtained from those peritoneal MC, and RT‐qPCR was utilized to analyze the expression of *Tnfa, Il6, Il1b, Cxcl1, Vegfa*, and *p21* mRNAs. MC sorted from peritoneal lavages of animals repeatedly treated with LPS displayed increased levels of pro‐inflammatory and pro‐angiogenic cytokine mRNAs, together with augmented levels of the senescence marker p21 (Figure 6I,J).

## 4 Discussion

Senescence is a process that occurs with distinct features in all immune cells (Zhao et al. 2024; Vicente et al. 2016). This change in phenotype leads to altered responses against antigens or tissue damage and can contribute to the age‐dependent decay in protective reactions and inflammaging. MC are long‐lived cells that seem to be subjected to very slow turnover (Blenkinsopp 1967; Padawer 1974; Chia et al. 2023; Tauber et al. 2023). Despite the information that exists in distinct immune cell populations, features of senescent MC are not well described.

Here we show that after prolonged culture, murine BMMC showed the expression of senescence markers, such as p16, p21, SA‐β‐Gal, a high number of cells arrested in the G1 phase of the cell cycle, and increased size and granularity. Also, they displayed high amounts of basal pro‐inflammatory and pro‐angiogenic cytokine mRNAs and a SASP characterized by IL‐23, TNF, IL‐6, VEGF, and CCL‐4. Accordingly with the International Cell Senescence Association (ICSA), and the recently published guidelines for minimal information on cellular senescence experimentation, a combination of more than two markers is a requisite to identify a cell as senescent (Ogrodnik et al. 2024; Gorgoulis et al. 2019). Our data show that a BMMC preparation composed of senescent cells can be obtained after 11 to 13 weeks of culture, indicating that long‐term cultures of BMMC can be utilized as a cellular model to analyze the effects of senescence in signaling events and effector functions in MC, together with the possible effects of senolytics and senomorphics in this cell model.

p16 expression was detected in the cell nucleus and cytoplasm of non‐senescent MC, whereas the total amount and nuclear localization of p16 increased in senescent ones. This is in line with observations made in other cell preparations. For example, p16 was detected in the cytoplasm of gastric cancer cells (Liu et al. 2009); it was also observable in cytoplasmic vesicles in response to distinct stressful conditions triggering autophagy in retinal pigment epithelial‐1 cells (Coryell et al. 2020) and p16 expression was detected in the nuclei and cytoplasm of senescent RAW 264.7 macrophages (Fang et al. 2024), suggesting that diverse patterns of p16 protein localization occur in senescent cells, probably related to the distinct important functions that this protein has been related to. For example, resistance to anoikis (Plath et al. 2000). On the other hand, cytoplasmic p21 expression was found related to higher migration and invasion abilities of cancer cells (Huang et al. 2014). According to that function, cytoplasmic p21 was reported to inhibit the activity of Rho kinase (ROCK) by forming a complex with that protein in Ras‐transformed cells, contributing to the loss of actin stress fibers and the decrease in cofilin phosphorylation, promoting migration (Lee and Helfman 2004). The increase of p21 in the cytosol of senescent BMMC could indicate improved migratory capacity, which coincides with an elevated basal and stimulated migration to S1P and LPS. Our data suggest a probable function of p21 in the control of the actin cytoskeleton in senescent MC.

SASP of senescent BMMC included the well‐recognized pro‐inflammatory cytokines (TNF, IL‐1β, and IL‐6) and the angiogenic factor VEGF. Depending on the produced amount, MC‐derived TNF produced in response to bacterial infections is associated with both protective and lethal responses in mice (Piliponsky et al. 2010; Echtenacher et al. 1996), suggesting that senescence alters the threshold between a beneficial and deleterious inflammatory response to Gram‐negative bacteria. Also, previous studies show that MC produce VEGF in response to monomeric IgE (mIgE), contributing to IgE‐stimulated angiogenesis in a murine model of tumor growth (Jiménez‐Andrade et al. 2013; Boesiger et al. 1998). Our results allow us to hypothesize that senescent MC could play an important role in tissue aging by exacerbating inflammation and vascular activity and probably promoting cell influx in tumors and tissues affected by autoimmune conditions. This hypothesis should be further explored in future research.

Metabolic changes have been reported associated with senescence in distinct cells (reviewed in Wiley and Campisi 2021; Martini and Passos 2023). Either high or lower mitochondrial activity has been observed in senescent cells. Our data showing that senescent MCs display higher basal OCR and higher activity of the mitochondrial respiratory chain are in line with other studies showing that senescence is accompanied by an increase in basal mitochondrial function. For example, in IMR90 fibroblasts, increased basal OCR and responsiveness to FCCP were observed after treatment with doxorubicin or decitabine, two compounds able to induce senescence (Kim et al. 2023). Also, in AML12 cells, senescence induced by hydrogen peroxide leads to increased OCR, ECAR, glycolysis, and high glycolytic reserve (Singh et al. 2020). It has been proposed that those characteristics observable in senescent cells could be attributable to an increase in the number of mitochondria per cell, since higher numbers of this organelle (although dysfunctional) have been observed in some senescent cells (reviewed in Miwa et al. 2022). Other authors have proposed that senescent cells can fully compensate for increased proton leakage by increasing electron‐transport activity and oxygen consumption in basal conditions (Hutter et al. 2004). Detailed analysis of mitochondrial function in senescent MC should be performed in the future to better understand age‐related metabolic changes and their consequences in MC‐related inflammation, since high metabolic activity observed in cells involved in active inflammatory reactions may contribute to a vicious cycle that accelerates aging and increases the risk of chronic diseases (Soto‐Heredero et al. 2020).

Our results show that senescence causes important modifications to FcεRI signaling, one of the most important activating pathways in MC (Nagata and Suzuki 2022). Also, altered TLR4 signaling, including increased NFκB basal phosphorylation and higher migration to LPS, was observed in senescent MC. Of note, *fcer1g*, the gene coding for the γ subunit of the FcεRI receptor, and the *ccl2* chemokine gene have been included in the top 20 significantly upregulated genes in the human and murine SASP clusters (Saul et al. 2022), suggesting that the observed elevated activity of senescent MC could importantly contribute to inflammaging.

Our data sum to the few studies that have analyzed changes in MC longevity. For example, in a study where BMMC cultures were maintained for 50 weeks, no alterations in IgE/Ag‐induced degranulation were found, although a significant decrease in Annexin V, together with an increase in the expression of Psme4/PA200 proteasomes was observed (Martin et al. 2019). Differences with our results could be attributed to the fact that cells used in the previous study were generated in media supplemented with 30% WEHI‐3B conditioned medium plus recombinant IL‐3. Since MC phenotype can rapidly change depending on growth factors present in the media and cells grown in WEHI‐conditioned media show distinct characteristics compared with those grown in the presence of recombinant IL‐3 (Razin et al. 1984), some differences could emerge between the two cell preparations.

Prolonged exposure to LPS promotes the expression of senescence markers in distinct cell types (Aquino‐Martinez et al. 2020; Kim et al. 2012), including macrophages (Fang et al. 2024; Danish et al. 2025). Our results showing that long‐term (5 days‐long) exposure to LPS leads to increased activity of β‐Gal, arrest in the G_1_ phase of the cell cycle, and a diminished number of cell divisions are in line with those studies and with others that have shown that long‐lasting stimulation of MC with pro‐inflammatory stimuli can induce phenotypic changes closely related to senescence. For example, in the human transformed MC line HMC‐1, RANKL reduces proliferation, inducing a senescent phenotype characterized by an increase in SAβ‐Gal activity and p53 and p21 expression in TSLP‐stimulated cells (Lim et al. 2022). On the other hand, in BMMCs from 6 to 12 weeks old, senescence‐associated parameters were not observed or were transiently detected with prolonged exposure to LPS. For example, p21 expression was elevated for a few days but finally diminished to basal levels and p16 protein was not significantly induced (although its mRNA tended to increase). Also, our data show that, after LPS removal, increased synthesis of key cytokines is observable 1 day after, but no accumulation is detected after that time (except for CCL2, where a tendency to increase up to 7 days was observable). Our data suggest that long‐term treatment with LPS promotes an acute senescence state in BMMC (Short et al. 2025).

Intraperitoneal secretion of TNF in response to Gram‐negative bacteria is a canonical MC‐dependent inflammatory reaction in mice (Malaviya et al. 1996; Echtenacher et al. 1996). That response can be mimicked in endotoxemia models, where LPS is *i.p*. administered in mice (Madera‐Salcedo et al. 2011). Our data from in vivo experiments show an increase in cytokine content in the peritoneum of aged mice and hyperresponsiveness after acute LPS challenge. Remarkably, as occurs in young animals, those responses were dependent on the presence of MC, as observed in MC‐deficient *c‐Kit* ^*Wsh/Wsh*^ and MC‐reconstituted *c‐Kit* ^*Wsh/Wsh*^ animals. Long‐term reconstitution of MC in the peritoneal cavity has been previously reported (Wu et al. 2023). In those studies, in vitro differentiated BMMC generated connective tissue MC with a long lifespan in vivo. Our data suggest that intraperitoneal MC from aged mice express a senescent phenotype characterized by higher basal levels of pro‐inflammatory cytokines and, importantly, an intense response after acute LPS challenge.

Our results showing an increased basal pro‐inflammatory activity of peritoneal MC in aged mice are in line with those studies showing some age‐related changes in MC function in other organs and species. For example, analyzing aged rat mesenteric lymphatic vessels, it was observed that there was a significant increase (close to 30%) in the number of MCs and a 400% increase in the number of activated MC when compared with young animals in resting conditions (Chatterjee and Gashev 2012). Remarkably, a diminished capacity for response in the presence of distinct stimuli was also observed in mesenteric vessel MC from aged individuals. Those authors concluded that pre‐activation of MC observed in aged animals contributed to the age‐associated impairment of lymphatic vessel function. In another study, the number of MC was analyzed in skin from young and aged rats (Abdel Hafez 2019). There, the authors found an increased number of total and activated MC in the skin of aged animals, suggesting that senescence increases basal production of pro‐inflammatory mediators, and that the secretory activity of dermal MC contributes to changes observed in aging skin. Also, increased activity of MC has been proposed as a marker of brown adipose tissue (BAT) aging (Mancini et al. 2021).

An increased population of cells in the G_1_ phase of the cell cycle and augmented p21 mRNA expression, together with high amounts of pro‐inflammatory and pro‐angiogenic cytokines, were observed in peritoneal MC recovered from chronic LPS‐treated animals, suggesting that, as observed in vitro, prolonged exposure to pro‐inflammatory stimuli promotes a stress‐induced senescence phenotype in MC and that this condition could contribute to the modified MC‐dependent innate immunity responses in vivo. Our results are similar to those showing higher MC activation after prolonged stress; for example, higher MC activation was correlated with frailty observed in aged individuals that presented long‐COVID. Patients with frailty showed a blood transcriptome with relevant disturbances in pathways regulating stress response, immune function, and metabolism, which were associated with MC activation (Li et al. 2023).

MC constitutes an important and ancient immune cell lineage that is responsible for sentinel functions, immunosurveillance, and the initiation of acute inflammatory reactions. Their remarkable capacity for the production of pro‐inflammatory and regulatory molecules able to activate endothelial cells and nerve terminals, together with variable but commonly long life inside tissues, places them in a central position in the control of inflammaging. Our study shows, for the first time, that senescence alters their basal and stimuli‐dependent responsiveness and cytokine secretion pattern of this cell type in vitro and in vivo, suggesting that aging promotes changes in local and systemic MC‐dependent reactions that could contribute to inflammaging.

## Author Contributions

I.‐S.A. and I.M.‐S. performed most of the experiments, analyzed results, and wrote the first draft of the manuscript; D.E.‐R. and M.‐M.P. performed selected experiments regarding the evaluation of protein phosphorylation in senescent cells and the effect of long‐term treatment of LPS on MC senescence; J.P. participated in the discussion of the results and experimental design; C.G.‐E. conceived and directed the study, was responsible for financing and experiment design, supervised all activities related to the project, and elaborated the final version of the manuscript.

## Disclosure

*Permission Statement*: All authors have read and agreed to the publication of this manuscript.

## Ethics Statement

All animal and experimental practices were approved by the Institutional Committee for the Care and Use of Laboratory Animals (CICUAL), Cinvestav, by the protocols No. 384‐24 and 378‐24.

## Conflicts of Interest

The authors declare no conflicts of interest.

## Supporting information


**Figure S1:** Gating strategy for flow cytometry assays. Sequential gating was applied to identify live bone marrow‐derived mast cells (BMMC) in flow cytometry. Total cells were gated first, followed by singlet selection and exclusion of dead cells using Ghost Dye R780 as a live/dead stain. Finally, FcεRI+ cells were gated to isolate live BMMC. Forward scatter height (FSC‐H) was used to analyze cell size and side scatter height (SSC‐H) to analyze cell complexity. Proliferation BMMC were quantified by gating Live FceRI+ on CTV to monitor the dilution of this tracer that occurs with cell division. Cell cycle analysis in live FcεRI+ cells was performed using Pyronin Y and Hoechst staining. TLR4 expression in BMMC was evaluated in gated live FcεRI+ cells.

**Figure S2:** Autofluorescence of non‐senescent and senescent BMMCs. Two‐way ANOVA, Tukey's multiple comparisons test. **p ≤ 0.01, ***p ≤ 0.001, ****p ≤ 0.0001 and ns = not significant.

**Figure S3:** Quantification of cytokine secretion in non‐stimulated BMMCs from cultures of 6, 8 and 12 weeks. One way ANOVA, Tukey's multiple comparisons test. *p ≤ 0.05, **p ≤ 0.01, ***p ≤ 0.005, ****p ≤ 0.001, *n* = 3.

**Figure S4:** Quantification of cytokine secretion in 1 h‐treated N‐Sc and Sc cells with vehicle or LPS (100 ng/mL). Two‐way ANOVA, Tukey's comparisons test *n* = 3, *p ≤ 0.05, **p ≤ 0.01, ***p ≤ 0.005, ****p ≤ 0.001.

**Figure S5:** Chronic treatment with LPS does not change BMMC viability or size and granularity. (A) The viability and (B) the size and complexity of BMMC chronically exposed to vehicle (Veh) or LPS (100 ng/mL) for 5 days, were assessed by flow cytometry. The gating strategy used for analysis is detailed in Figure S1. Bars represent mean ± SEM, *n* = 3, *t*‐test, *p ≤ 0.05.

**Figure S6:** Quantification of cytokine secretion in response to chronic treatment with LPS. Non‐senescent BMMCs were treated with vehicle or LPS (100 ng/mL) for 5 days and cytokine secretion was analyzed. One way ANOVA, Tukey's multiple comparisons test. *p ≤ 0.05, **p ≤ 0.01, *n* = 3.

**Figure S7:** Flow cytometry gating strategy used to identify peritoneal mast cells. The light scatter profile for cells was analyzed based on forward scatter (FSC‐A) and side scatter (SSC‐A), with the region set to distinguish granulocytes. Singlets gating was then applied based on FSC‐A vs. FSC‐H and then SSC‐A vs. SSC‐H, respectively. Following this, cells were classified as live (ghost dye negative) CD45+ cells. From live CD45+ population, MC were selected based on the expression of FcεRI+ and c‐Kit+.

**Table S1:** acel70186‐sup‐0008‐TableS1.pdf.

## Data Availability

The data that support the findings of this study are available on request from the corresponding author.
