# Supplementary figures and images for: A Prominent Pro‐Inflammatory Phenotype Is Observed in Replication and Stress‐Induced Senescent Mast Cells
Source: Aging Cell. 2025 Aug 28;24(10):e70186. doi: 10.1111/acel.70186 (PMC12507405; doi:10.1111/acel.70186)

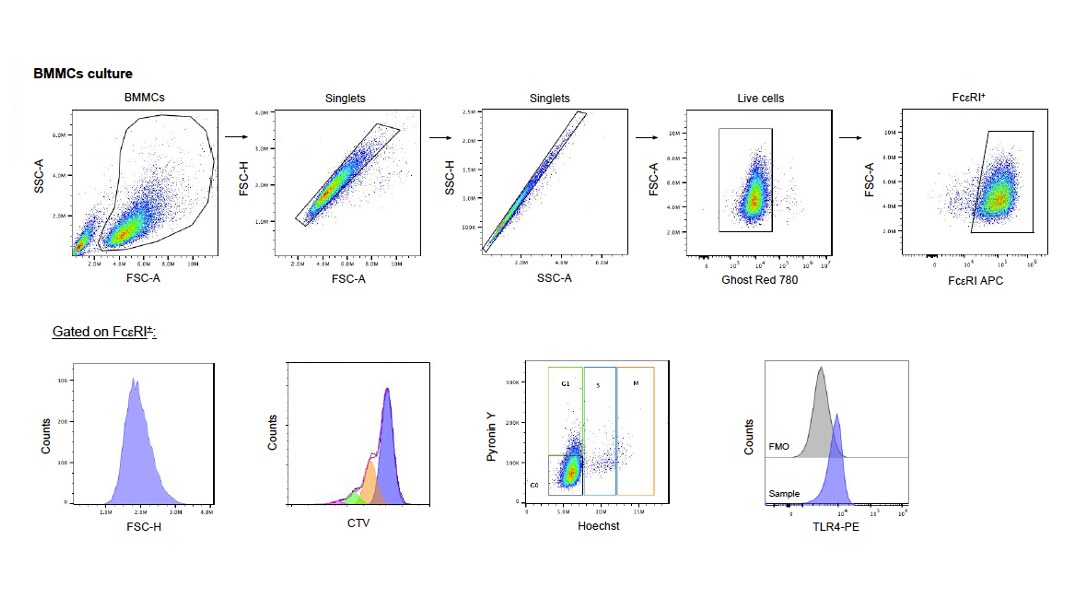

Supplement: Supplementary file 1 — Figure S1: Gating strategy for flow cytometry assays. Sequential gating was applied to identify live bone marrow‐derived mast cells (BMMC) in flow cytometry. Total cells were gated first, followed by singlet selection and exclusion of dead cells using Ghost Dye R780 as a live/dead stain. Finally, FcεRI+ cells were gated to isolate live BMMC. Forward scatter height (FSC‐H) was used to analyze cell size and side scatter height (SSC‐H) to analyze cell complexity. Proliferation BMMC were quantified by gating Live FceRI+ on CTV to monitor the dilution of this tracer that occurs with cell division. Cell cycle analysis in live FcεRI+ cells was performed using Pyronin Y and Hoechst staining. TLR4 expression in BMMC was evaluated in gated live FcεRI+ cells. [file ACEL-24-e70186-s002.tiff]

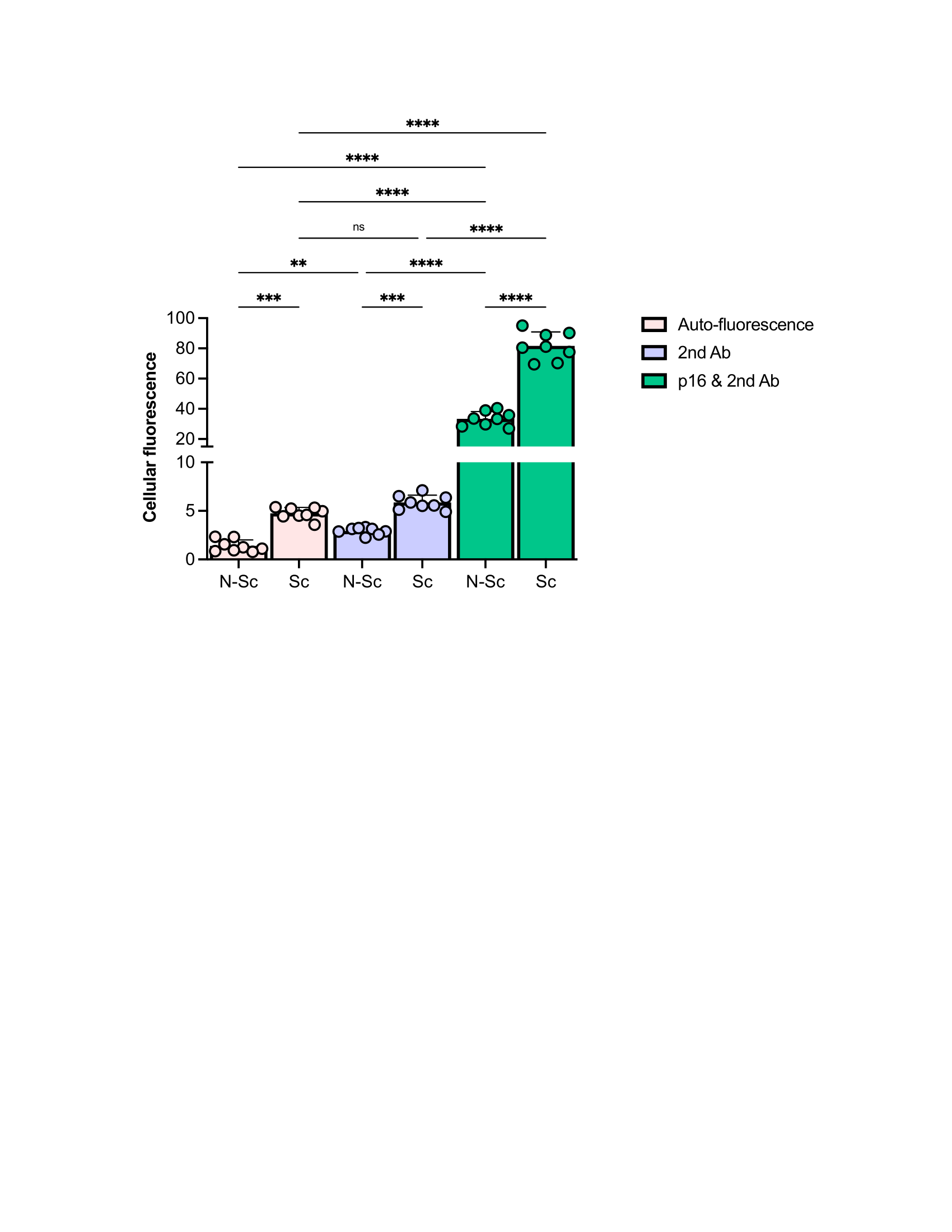

Supplement: Supplementary file 2 — Figure S2: Autofluorescence of non‐senescent and senescent BMMCs. Two‐way ANOVA, Tukey's multiple comparisons test. **p ≤ 0.01, ***p ≤ 0.001, ****p ≤ 0.0001 and ns = not significant. [file ACEL-24-e70186-s004.tiff]

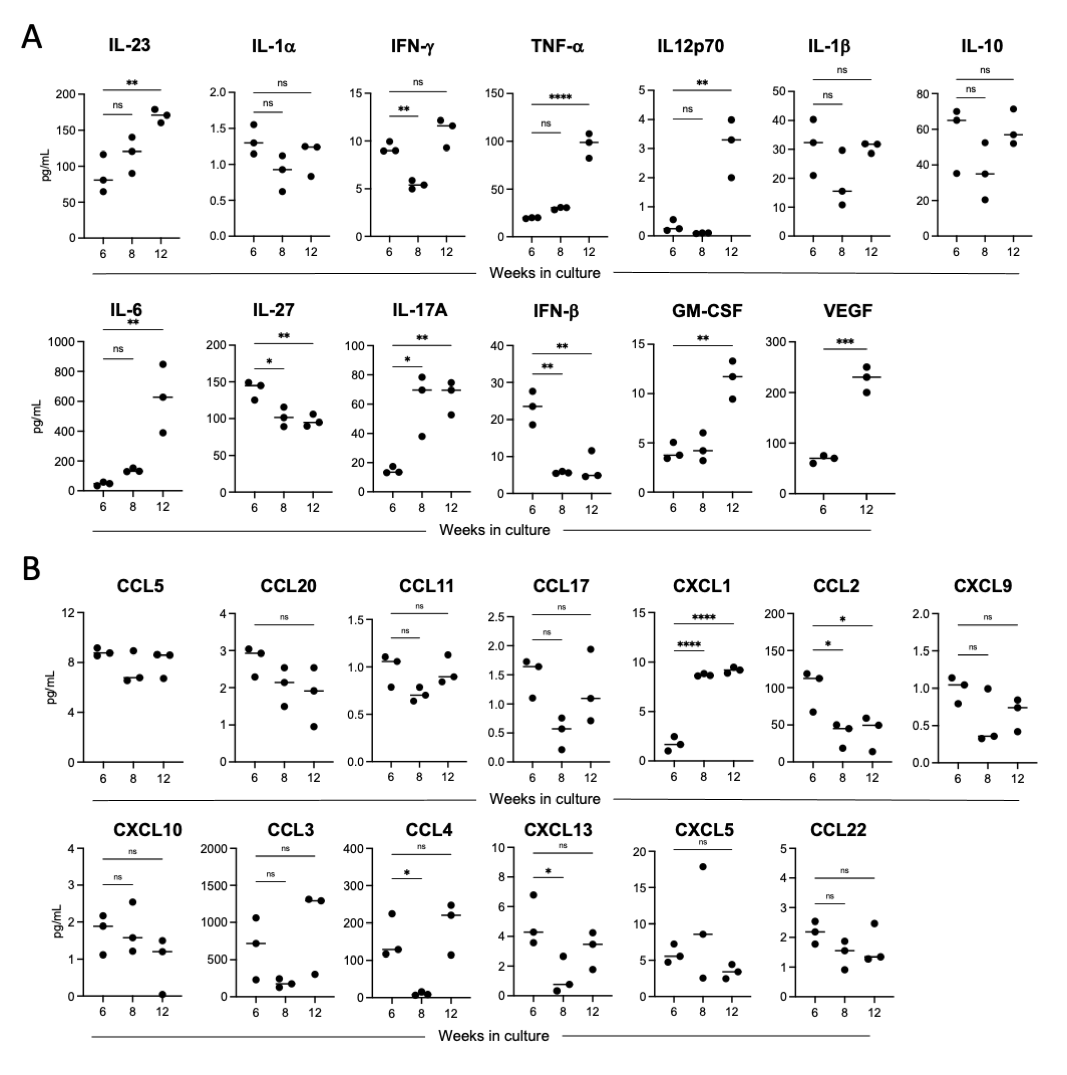

Supplement: Supplementary file 3 — Figure S3: Quantification of cytokine secretion in non‐stimulated BMMCs from cultures of 6, 8 and 12 weeks. One way ANOVA, Tukey's multiple comparisons test. *p ≤ 0.05, **p ≤ 0.01, ***p ≤ 0.005, ****p ≤ 0.001, n = 3. [file ACEL-24-e70186-s005.tiff]

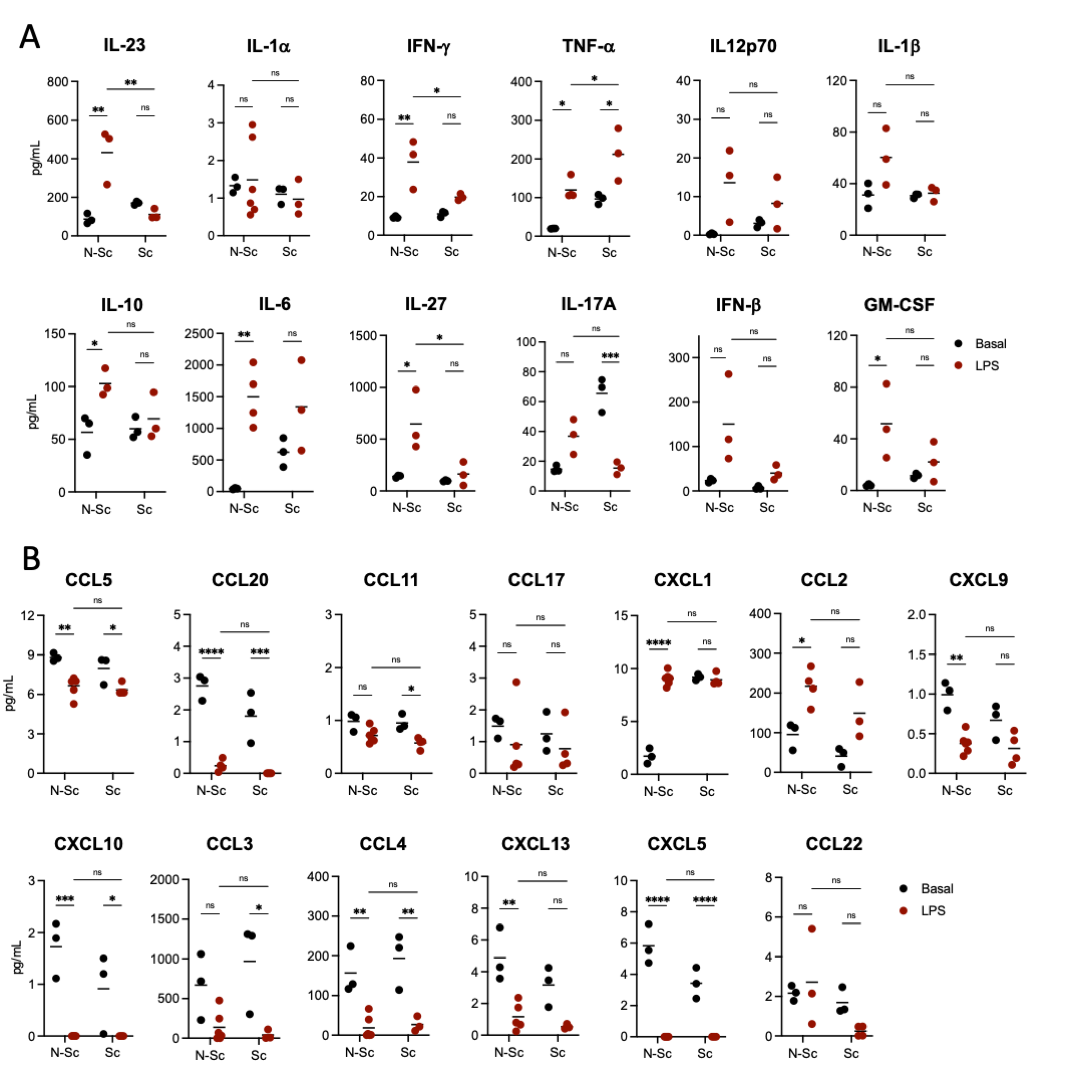

Supplement: Supplementary file 4 — Figure S4: Quantification of cytokine secretion in 1 h‐treated N‐Sc and Sc cells with vehicle or LPS (100 ng/mL). Two‐way ANOVA, Tukey's comparisons test n = 3, *p ≤ 0.05, **p ≤ 0.01, ***p ≤ 0.005, ****p ≤ 0.001. [file ACEL-24-e70186-s003.tiff]

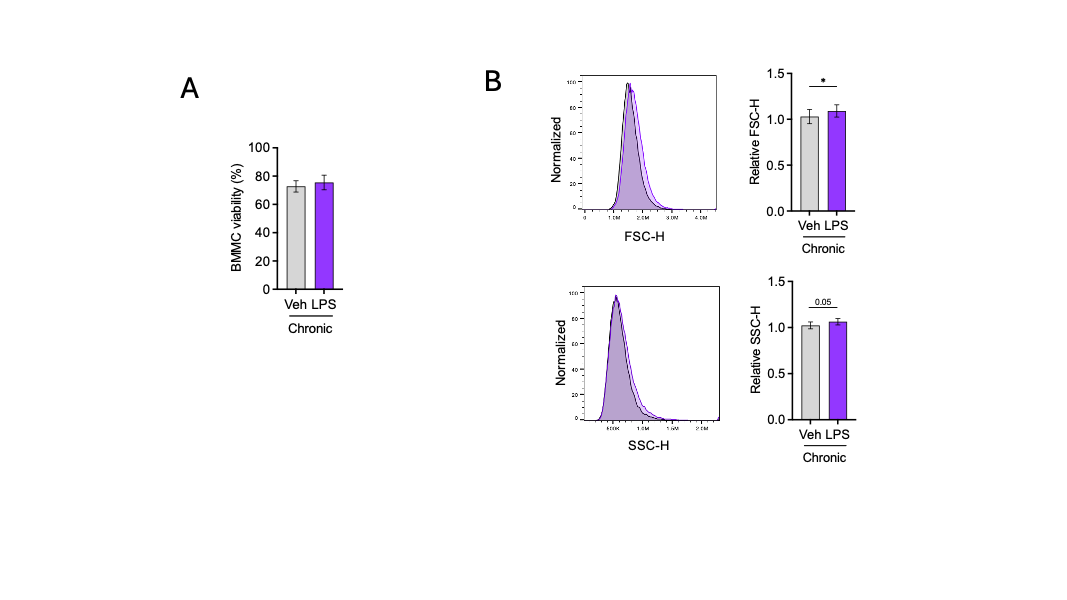

Supplement: Supplementary file 5 — Figure S5: Chronic treatment with LPS does not change BMMC viability or size and granularity. (A) The viability and (B) the size and complexity of BMMC chronically exposed to vehicle (Veh) or LPS (100 ng/mL) for 5 days, were assessed by flow cytometry. The gating strategy used for analysis is detailed in Figure S1. Bars represent mean ± SEM, n = 3, t‐test, *p ≤ 0.05. [file ACEL-24-e70186-s007.tiff]

**A**

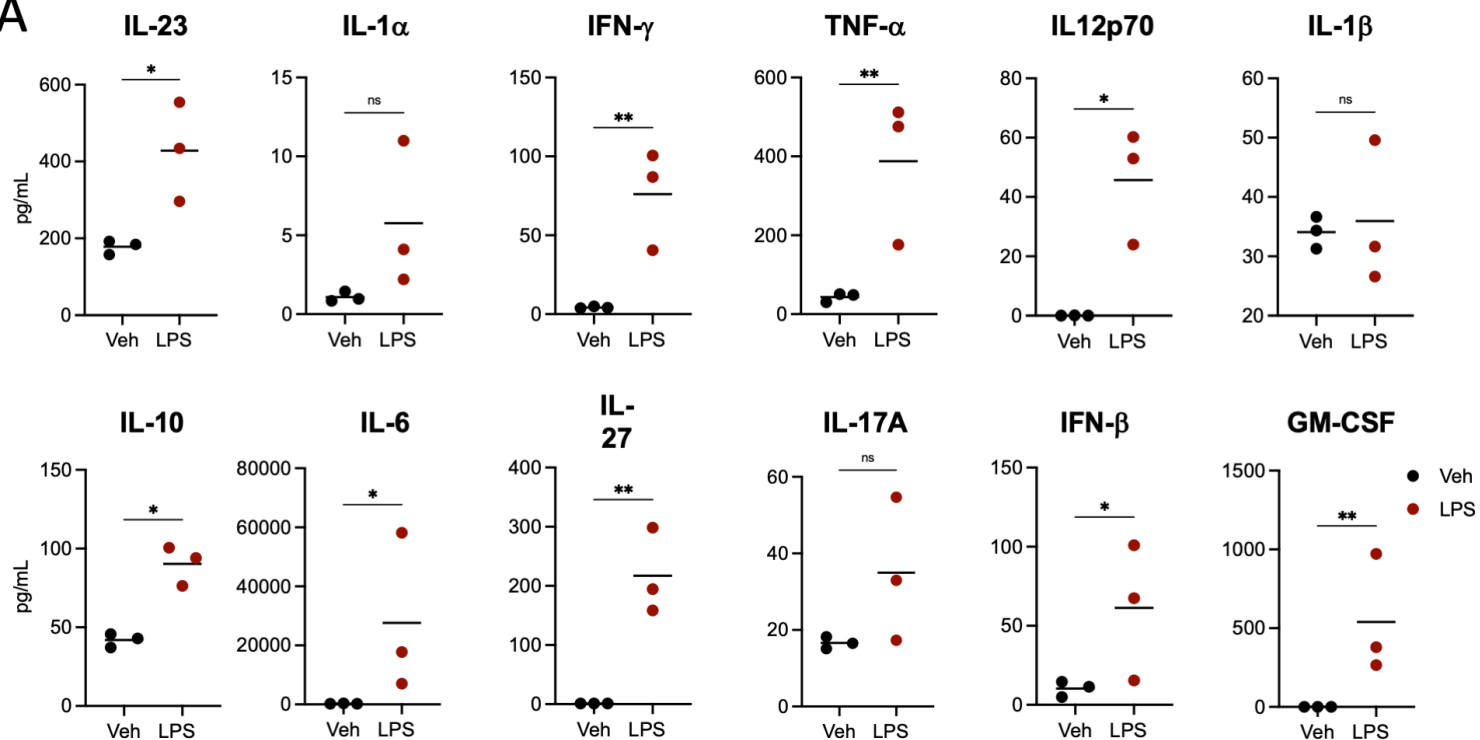

**B**

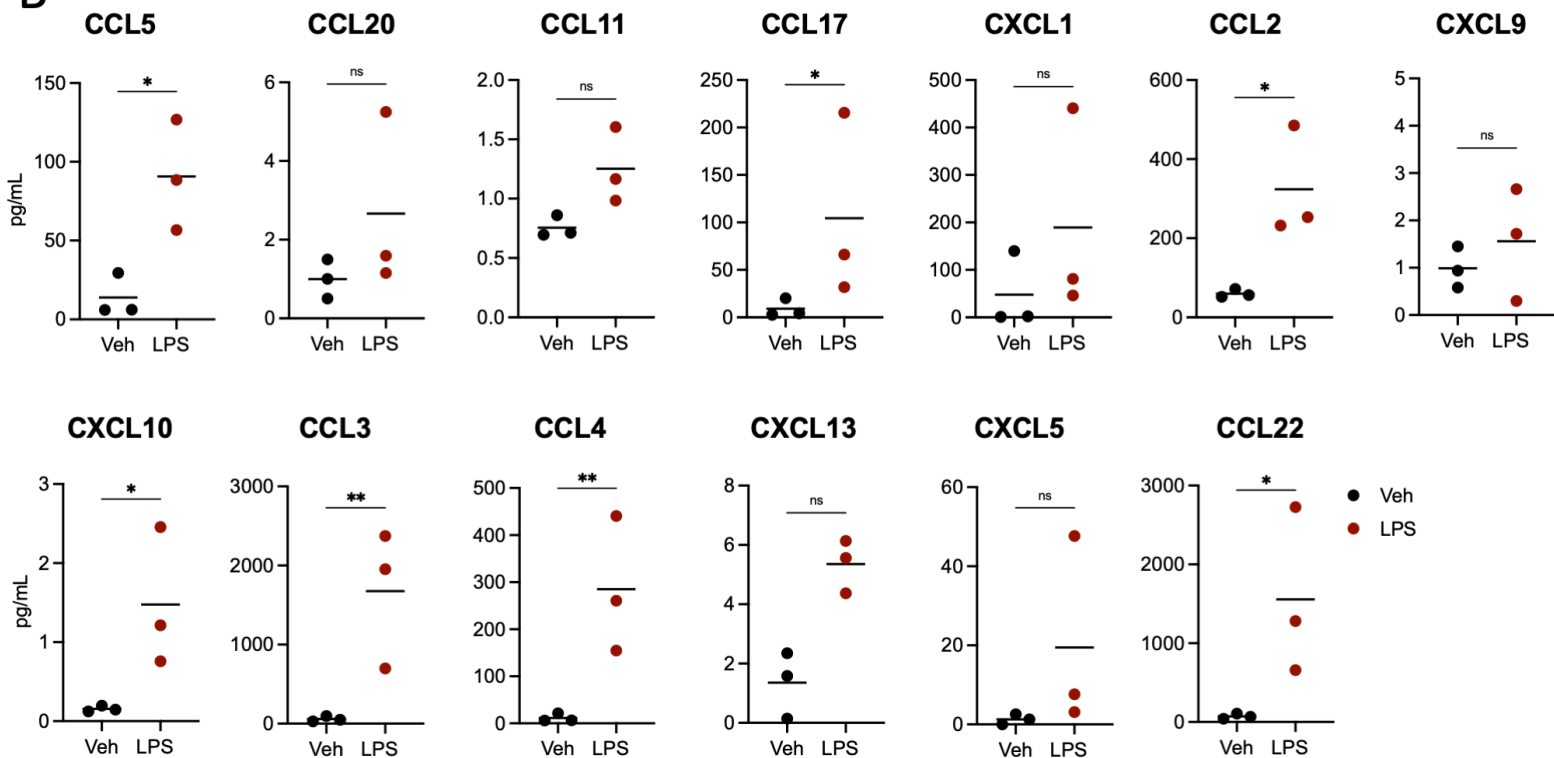

Supplement: Supplementary file 6 — Figure S6: Quantification of cytokine secretion in response to chronic treatment with LPS. Non‐senescent BMMCs were treated with vehicle or LPS (100 ng/mL) for 5 days and cytokine secretion was analyzed. One way ANOVA, Tukey's multiple comparisons test. *p ≤ 0.05, **p ≤ 0.01, n = 3. [file ACEL-24-e70186-s008.pdf]

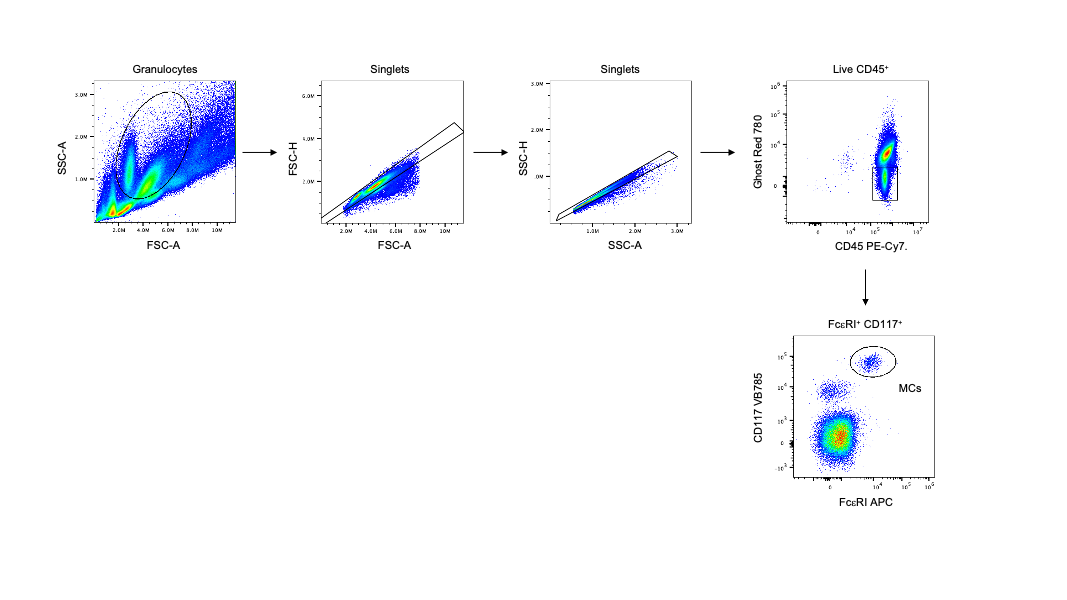

Supplement: Supplementary file 7 — Figure S7: Flow cytometry gating strategy used to identify peritoneal mast cells. The light scatter profile for cells was analyzed based on forward scatter (FSC‐A) and side scatter (SSC‐A), with the region set to distinguish granulocytes. Singlets gating was then applied based on FSC‐A vs. FSC‐H and then SSC‐A vs. SSC‐H, respectively. Following this, cells were classified as live (ghost dye negative) CD45+ cells. From live CD45+ population, MC were selected based on the expression of FcεRI+ and c‐Kit+. [file ACEL-24-e70186-s006.tiff]
