# Supplementary material for: A Prominent Pro‐Inflammatory Phenotype Is Observed in Replication and Stress‐Induced Senescent Mast Cells
Source: Aging Cell. 2025 Aug 28;24(10):e70186. doi: 10.1111/acel.70186 (PMC12507405; doi:10.1111/acel.70186)
Supplement: Supplementary file 8 — Table S1: acel70186‐sup‐0008‐TableS1.pdf. [file ACEL-24-e70186-s001.pdf]

Suppl. Table I. Statistical Analysis of Data presented on Figures 6B and 6C

TNF production

Age (weeks)	Group Number	Mouse Strain	Condition	Mean ± SEM (pg/mL)	Significance vs Group 1	Significance vs Group 2	Significance vs Group 3	Significance vs Group 4	Significance vs Group 5	Significance vs Group 6	Significance vs Group 7	Significance vs Group 8	Significance vs Group 9	Significance vs Group 10	Significance vs Group 11	Significance vs Group 12
8	1	C57BL6/J	Saline	105.3 ± 8.7	NA	****	NS	NS	NS	****	*	****	*	****	**	****
	2	C57BL6/J	LPS	408.8 ± 30.2	****	NA	****	****	****	NS	****	****	***	NS	*	****
	3	c-Kit Wsh/Wsh	Saline	60.3 ± 6.7	NS	****	NA	NS	NS	****	**	****	**	****	****	****
	4	c-Kit Wsh/Wsh	LPS	80.0 ± 5.0	NS	****	NS	NA	NS	****	*	****	**	****	***	****
	5	c-Kit Wsh/Wsh Rec	Saline	80.3 ± 6.9	NS	****	NS	NS	NA	****	*	****	**	****	***	****
	6	c-Kit Wsh/Wsh Rec	LPS	459.0 ± 22.7	****	NS	****	****	****	NA	****	****	****	*	***	****
60	7	C57BL6/J	Saline	213.5 ± 16.7	*	****	**	*	*	****	NA	****	NS	NS	NS	****
	8	C57BL6/J	LPS	822.5 ± 48.7	****	****	****	****	****	****	****	NA	****	****	****	****
	9	c-Kit Wsh/Wsh	Saline	229.5 ± 18.4	*	***	**	**	**	****	NS	****	NA	NS	NS	****
	10	c-Kit Wsh/Wsh	LPS	321.5 ± 15.6	****	NS	****	****	****	*	NS	****	NS	NA	NS	****
	11	c-Kit Wsh/Wsh Rec	Saline	270.5 ± 10.8	**	*	****	***	***	***	NS	****	NS	NS	NA	****
	12	c-Kit Wsh/Wsh Rec	LPS	1143.3 ± 46.1	****	****	****	****	****	****	****	****	****	****	****	NA

IL-6 production

Age (weeks)	Group Number	Mouse Strain	Condition	Mean ± SEM (pg/mL)	Significance vs Group 1	Significance vs Group 2	Significance vs Group 3	Significance vs Group 4	Significance vs Group 5	Significance vs Group 6	Significance vs Group 7	Significance vs Group 8	Significance vs Group 9	Significance vs Group 10	Significance vs Group 11	Significance vs Group 12
8	1	C57BL6/J	Saline	106.5 ± 6.9	NA	****	NS	****	NS	****	*	****	NS	****	*	****
	2	C57BL6/J	LPS	494.5 ± 10.8	****	NA	****	*	****	NS	****	****	****	NS	****	****
	3	c-Kit Wsh/Wsh	Saline	49.0 ± 5.2	NS	****	NA	****	NS	****	***	****	*	****	***	****
	4	c-Kit Wsh/Wsh	LPS	380.3 ±12.2	****	*	****	NA	****	**	****	****	****	***	****	****
	5	c-Kit Wsh/Wsh Rec	Saline	110.3 ± 10.4	NS	****	NS	****	NA	****	NS	****	NS	****	NS	****
	6	c-Kit Wsh/Wsh Rec	LPS	505.5 ± 5.7	****	NS	****	**	****	NA	****	****	****	NS	****	***
60	7	C57BL6/J	Saline	215.0 ± 8.2	*	****	***	****	NS	****	NA	****	NS	****	NS	****
	8	C57BL6/J	LPS	864.3 ± 50.4	****	****	****	****	****	****	****	NA	****	****	****	NS
	9	c-Kit Wsh/Wsh	Saline	156.2 ± 7.5	NS	****	*	****	NS	****	NS	****	NA	****	NS	****
	10	c-Kit Wsh/Wsh	LPS	526.5 ± 14.6	****	NS	****	***	****	NS	****	****	****	NA	****	****
	11	c-Kit Wsh/Wsh Rec	Saline	209.5 ± 9.7	*	****	***	****	NS	****	NS	****	NS	****	NA	****
	12	c-Kit Wsh/Wsh Rec	LPS	885.5 ± 42.4	****	****	****	****	****	***	****	NS	****	****	****	NA

NA=The determination does Not Apply	NS = not significant	* $p \leq 0.05$	** $p \leq 0.01$	*** $p \leq 0.001$	**** $p \leq 0.0001$
